# A gut‐centric view of aging: Do intestinal epithelial cells contribute to age‐associated microbiota changes, inflammaging, and immunosenescence?

**DOI:** 10.1111/acel.13700

**Published:** 2022-08-24

**Authors:** Leah S. Hohman, Lisa C. Osborne

**Affiliations:** ^1^ Department of Microbiology & Immunology, Life Sciences Institute University of British Columbia Vancouver British Columbia Canada

**Keywords:** aging, inflammaging, intestinal epithelial cells, immunosenescence, microbiome

## Abstract

Intestinal epithelial cells (IECs) serve as both a physical and an antimicrobial barrier against the microbiota, as well as a conduit for signaling between the microbiota and systemic host immunity. As individuals age, the balance between these systems undergoes a myriad of changes due to age‐associated changes to the microbiota, IECs themselves, immunosenescence, and inflammaging. In this review, we discuss emerging data related to age‐associated loss of intestinal barrier integrity and posit that IEC dysfunction may play a central role in propagating age‐associated alterations in microbiota composition and immune homeostasis.

## Graphical abstract

Age‐related comorbidities are associated with changes in the immune system, including inflammaging and immunosenesence. In this review, the authors discuss the interplay between the immune system, the microbiota, and intestinal epithelial cells. Intestinal epithelial cells remain an understudied area in the aging process, and enhanced understanding of how these cell types coordinate signals between the microbiota and the immune system may offer insights into novel strategies to promote healthy aging.

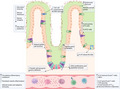

## 1 AGE‐RELATED ALTERATIONS ARE A GROWING HEALTH CONCERN

The global population is aging, and the World Health Organization (WHO) projects that the number of individuals aged 60 and older will double by 2050, while the number of individuals aged 80 and over will triple between 2020 and 2050. The increasing age of the global population is associated with significant consequences and costs for both societal and health infrastructure. Aging is associated with a host of health complications, including cancer and metabolic, cardiovascular, and neurodegenerative disorders (Deleidi et al., 2015; Franceschi et al., 2018; Goronzy & Weyand, 2012), yet not all individuals age equally, leading to the hypothesis that environmental and genetic factors conspire to promote healthy or unhealthy aging. Unraveling the qualities that promote healthy over unhealthy aging represents an important research question, which may translate into significant improvements in the quality of life for the global aging population and the rates of multifactorial age‐associated comorbidities.

While the biomedical research enterprise focuses on understanding mechanisms to enhance healthy aging and longevity, there are coincident societal implications. Aged individuals can provide valuable societal contributions through their accumulated knowledge and generational insights, yet this wisdom is commonly overlooked in Western societies, resulting in increasing isolation of elders. Ageism has been recognized as a global challenge by WHO with an estimated 6.3 million cases of depression thought to be attributable to ageism. Further, ageism is associated with worsened physical and mental health, increased loneliness and isolation, financial insecurity, decreased quality of life, and premature death. A shift in these views and the perception of the role of the elderly in society will be necessary to ensure that the idea of “healthy aging” holistically considers quality of life and continued societal integration and inclusion, as well as disease‐free lifespan.

Common age‐associated comorbidities are intimately tied to immune dysregulation. In aged individuals (generally >65 years old for humans, >20 months old for C57BL/6 mice), the immune system undergoes characteristic changes. First is the development of inflammaging, a state of chronic, systemic, sterile, low‐level inflammation (Franceschi et al., 2000) that is associated with increased susceptibility to the development of metabolic, cardiovascular, and neurodegenerative disorders. Paradoxically, a concomitant process known as immunosenescence, which is characterized by dampened innate and adaptive immune responses to antigen challenge, underlies age‐associated deficits in vaccine efficacy and response to infection. Age‐related immunosenescence may also contribute to cancer risk as a consequence of suboptimal immunosurveillance.

**FIGURE 1:**
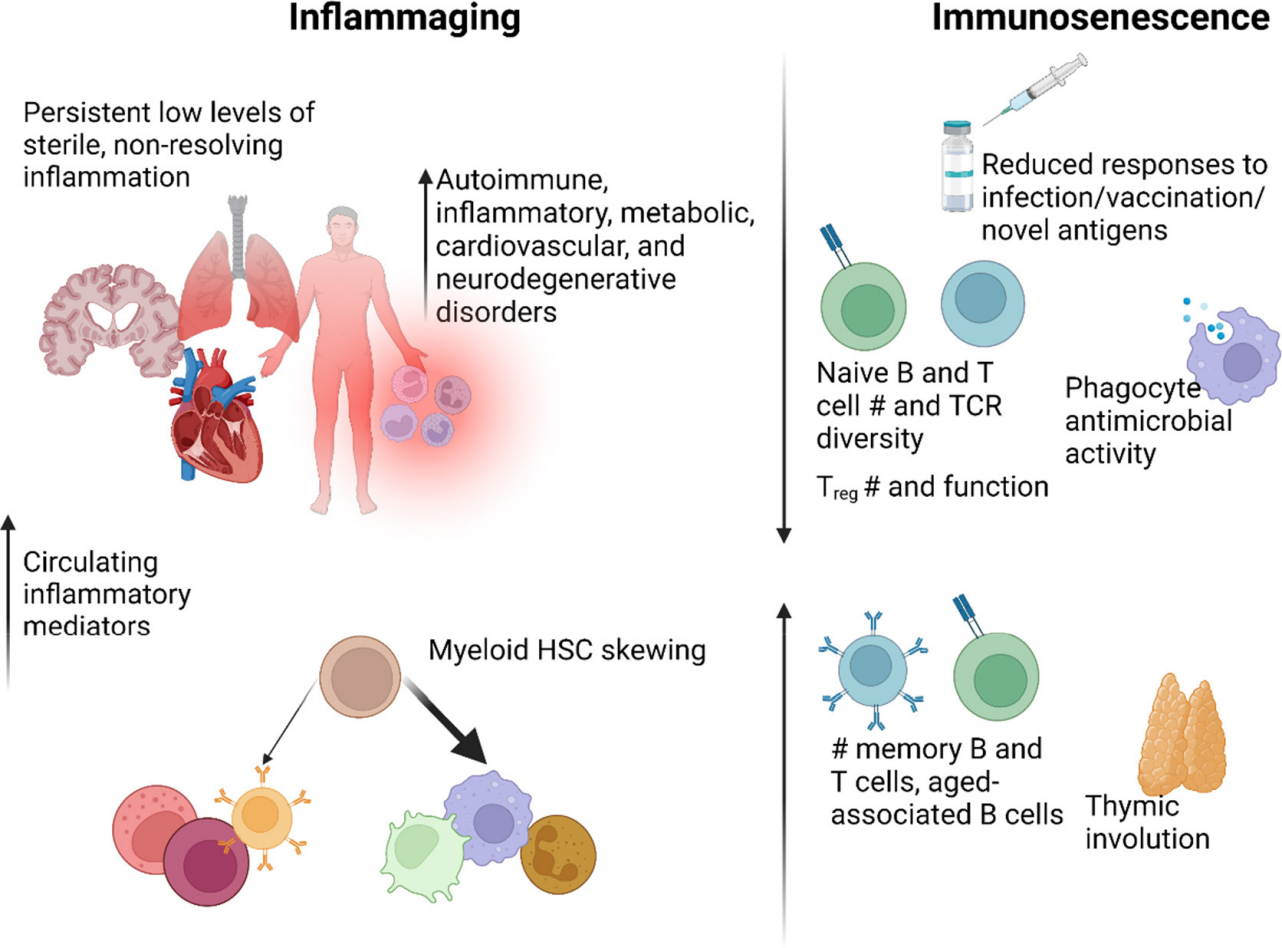
Hallmarks of inflammaging and immunosenescense in both humans and mice

Age‐related alterations of the adaptive immune system include decreased B cell diversity, accumulation of age‐associated B cells, and decreased antibody responses following vaccination; thymic involution, resulting in reduced rates of lymphopoiesis, fewer naïve T cells, and a less diverse T cell repertoire; and elevated numbers of hyporesponsive T cells expressing the inhibitory receptors Tim‐3 and PD‐1 (Agrawal et al., 2007; Appay & Sauce, 2014; Channappanavar et al., 2009; Ciabattini et al., 2018; Decman et al., 2012; Dunn‐Walters, 2016; Frasca & Blomberg, 2009; Gibson et al., 2009; Gustafson et al., 2020; Lee et al., 2016; Ma et al., 2019; Pang et al., 2011; Rossi et al., 2005; Shimada et al., 2009; Thomas et al., 2020). Aging is also associated with profound functional changes in innate immune cells, including altered dendritic cell function, decreased phagocyte antimicrobial activity, reduced chemokine/cytokine production in response to damage‐associated molecular pattern (DAMP)/microbe‐associated molecular pattern (MAMP) stimuli, and downstream consequences in adaptive immune priming and function (Boehmer et al., 2005; Liang et al., 2009; Renshaw et al., 2002; Shaik‐Dasthagirisaheb et al., 2010; Shaw et al., 2013; Toapanta & Ross, 2009). For the purposes of this review, we have compiled a list of key characteristics of immunosenescence and inflammaging that are common in both mice and humans in Figure 1.

In recent years, multiple studies have begun to untangle the profound impact of the microbiota on host physiology, including the immune system. In humans (Odamaki et al., 2016; Ragonnaud & Biragyn, 2021; Xu et al., 2019), mice (Boehme et al., 2021; Fransen et al., 2017; Langille et al., 2014; Thevaranjan et al., 2017), and flies (Broderick et al., 2014; Clark et al., 2015; Shukla et al., 2021), age‐related shifts in the composition of the microbiota have been associated with immune consequences and complications. The conservation of these phenotypes has led to the proposal that the modifiable nature of the microbiota may be exploited to limit detrimental consequences of immunosenescence or inflammaging (Biagi et al., 2016; Bosco & Noti, 2021; Ragonnaud & Biragyn, 2021; Xu et al., 2019).

The gut microbiota of otherwise healthy people over 65 years of age often significantly differs compared with younger adults, and reduced α‐diversity is a common feature (Bosco & Noti, 2021; Collino et al., 2013; Luan et al., 2020; Wu et al., 2019). These shifts in microbial community composition have been associated with frailty, altered immune signaling, and modified intestinal barrier integrity (Bosco & Noti, 2021; Claesson et al., 2012; Jackson et al., 2016; Magrone & Jirillo, 2013; Ragonnaud & Biragyn, 2021). Notably, centenarians (99–104 years old) and semi‐supercentenarian (105–109 years old) exhibit unique hallmark changes to the microbiota, such as increased α‐diversity, an association that suggests an intimate link between improved longevity and the microbiota (Biagi et al., 2016; Wilmanski et al., 2021).

Intestinal epithelial cells (IECs) are a diverse population of epithelial cells that form a continuous barrier separating the external environment (the intestinal lumen) from the underlying tissue. As such, they serve as a critical interface between the microbiota and the rest of the host immune system. However, we have limited understanding of how age impacts the function of the multiple lineages of IECs that form the interface between our immune system and the microbe‐rich intestinal lumen. In this review, we will provide an overview of age‐related changes to the microbiota, IECs, and the immune system and propose that altered IEC function with age is central to the dysregulation of this tripartite interaction in aging individuals.

## 2 AGE‐RELATED ALTERATIONS TO THE MICROBIOTA AND ITS METABOLITES

### 2.1 Age‐associated shifts in microbial composition

The intestinal multibiome is a highly diverse community of microbes and eukaryotes, including bacteria, archaea, viruses, fungi, protists, and sometimes helminths that exist within a host (Belkaid & Hand, 2014; Filyk & Osborne, 2016). While in this review we will focus on bacterial components of the microbiota, the potential impacts of other members of the gut multibiome are often understudied, yet important, contributors to health outcomes. The community composition of the microbiota is impacted by a plethora of individual and environmental factors including geography, immunological exposure, infection history, medical history (type of birth, antibiotic use, etc), age, sex, and genetics.

Interestingly, successful aging and centenarian status is associated with a unique microbiome compared to adult controls. Unlike standard elderly populations, which generally exhibit dysbiosis, decreased microbial diversity, and increased representation of proteolytic bacteria (Woodmansey, 2007), individuals with improved longevity (>80 years) tend to harbor a microbiome characterized by depletion of core bacteria and increased α‐diversity (Biagi et al., 2016; Wilmanski et al., 2021). For example, *Akkermansia*, *Bifidobacterium*, *Alistipes*, and *Christensenellaceae* (Biagi et al., 2016; Drago et al., 2012; Ragonnaud & Biragyn, 2021; Tuikhar et al., 2019 *)* are enriched in centenarian and semi‐supercentenarian humans relative to both younger adults and elderly individuals, suggesting that these microbial community members may be uniquely associated with successful aging. *Akkermansia*, in particular, has been linked to the production of mucin and maintenance of intestinal barrier integrity in aged murine models (Bodogai et al., 2018), as well improved longevity and healthspan following oral gavage in short‐lived progeroid mice (Bárcena et al., 2019) and increased production of antiaging‐associated polyamines and short‐chain fatty acids (SCFAs) (Grajeda‐Iglesias et al., 2021). Additionally, *Christensenellaceae* has been identified as one of the most heritable members of the microbial community, suggesting that there may be a genetic component to the acquisition of a successful aging/centenarian microbiota (Goodrich et al., 2014). This is further supported by the finding that siblings of centenarians have a life‐long mortality advantage, though this study did not examine microbiome composition (Perls et al., 2002).

### 2.2 Microbiota‐derived metabolites in aging

Going beyond the structural organization of microbial communities, understanding the functional effects of microbial‐derived metabolites is critical for enhanced understanding how host–microbiota interactions influence host health. In addition to the increased translocation of bacterial products seen in aged mice that could contribute to inflammaging, continually emerging evidence that bacterial‐derived metabolites directly influence host cell function (including IECs and immune cells) suggests that age‐associated immune dysregulation and associated comorbidities may be influenced by these molecules (Parker et al., 2022; Thevaranjan et al., 2017). As an example, microbiota‐derived SCFAs exert wide‐ranging effects on the intestinal environment, including promoting intestinal barrier integrity and mucus production, regulating and supporting commensal microbes, and mediating immune tolerance in the gut, including T regulatory cell (T_reg_) homeostasis (Corrêa‐Oliveira et al., 2016; Smith et al., 2013). Despite evidence that SCFAs decline in the standard aged individual due to a shift from saccharolytic fermentation to more proteolytic, pro‐inflammatory activities (Salazar et al., 2019), centenarian microbiomes have been noted to have a high capacity for central metabolism, including glycolysis and production of SCFAs (Wu et al., 2019).

Increased representation of proteolytic bacteria is a common feature in aging microbiomes, which could be predictive of elevated levels of the tryptophan‐derived metabolite indole. Counterintuitively, indole levels have been reported to decrease with age in mouse and human fecal samples and mouse serum (Ruiz‐Ruiz et al., 2020; Wu et al., 2021). Instead, in both aged mice and nonagenarians, kynurenine, an alternative tryptophan‐derived product is elevated, and increased levels of kynurenine predicted mortality in nonagenarians, suggesting that diversion of tryptophan from indole toward kynurenine may negatively impact healthspan in aged individuals (Pertovaara et al., 2006; Wu et al., 2021). Evidence that commensal microbiota‐derived indoles can increase healthspan in multiple organisms including *Caenorhabditis elegans*, *Drosophila melanogaster*, and mice (Sonowal et al., 2017) suggests that dietary supplementation with indole or alternative promotion of indole‐producing bacteria in the microbiota may result in improved health outcomes for aged individuals. Indeed, indole‐3‐carbinol supplementation has been found to improve *Clostridium difficile* outcomes in mice (Julliard et al., 2017), and colonization of young and aged mice with indole secreting bacteria can promote epithelial cell proliferation (Powell et al., 2020), supporting a critical role for indole in the modulation of aged‐associated changes to mucosal immunity and IEC renewal. Locally, metabolites impact IECs, which also undergo a variety of age‐related changes. Whether these IEC changes are age‐intrinsic and contribute to selecting an altered microbiota or are driven by an altered microbiome and microbial metabolites with age remains unknown, as does the relative importance of IEC function in age‐related immune dysregulation.

Metabolites are promising candidates for the translation of intestinal signals and regulation of distal cellular function, as well as control of persistent, low‐grade inflammation. Notably, the biosynthetic pathways of secondary bile acids, which have potent antimicrobial activity against Gram‐positive pathogens, are enriched in the centenarian microbiome (Sato et al., 2021). Secondary bile acids represent an intriguing area of potential study for successful versus unsuccessful aging as they have notable and predominantly anti‐inflammatory, enhanced barrier integrity, and pro‐wound repair effects on both IEC and immune cell populations (Sun et al., 2021). However, given the multitude of factors at play in the development and progression of the aged gut microbial community, there is likely not one “panacea” gut microbial composition, but rather a balance of diversity and evenness of microbial members and their metabolites that supports healthy aging and immune homeostasis (Rampelli et al., 2016).

### 2.3 Microbiota, metabolites, and age‐associated immune effects

**FIGURE 2:**
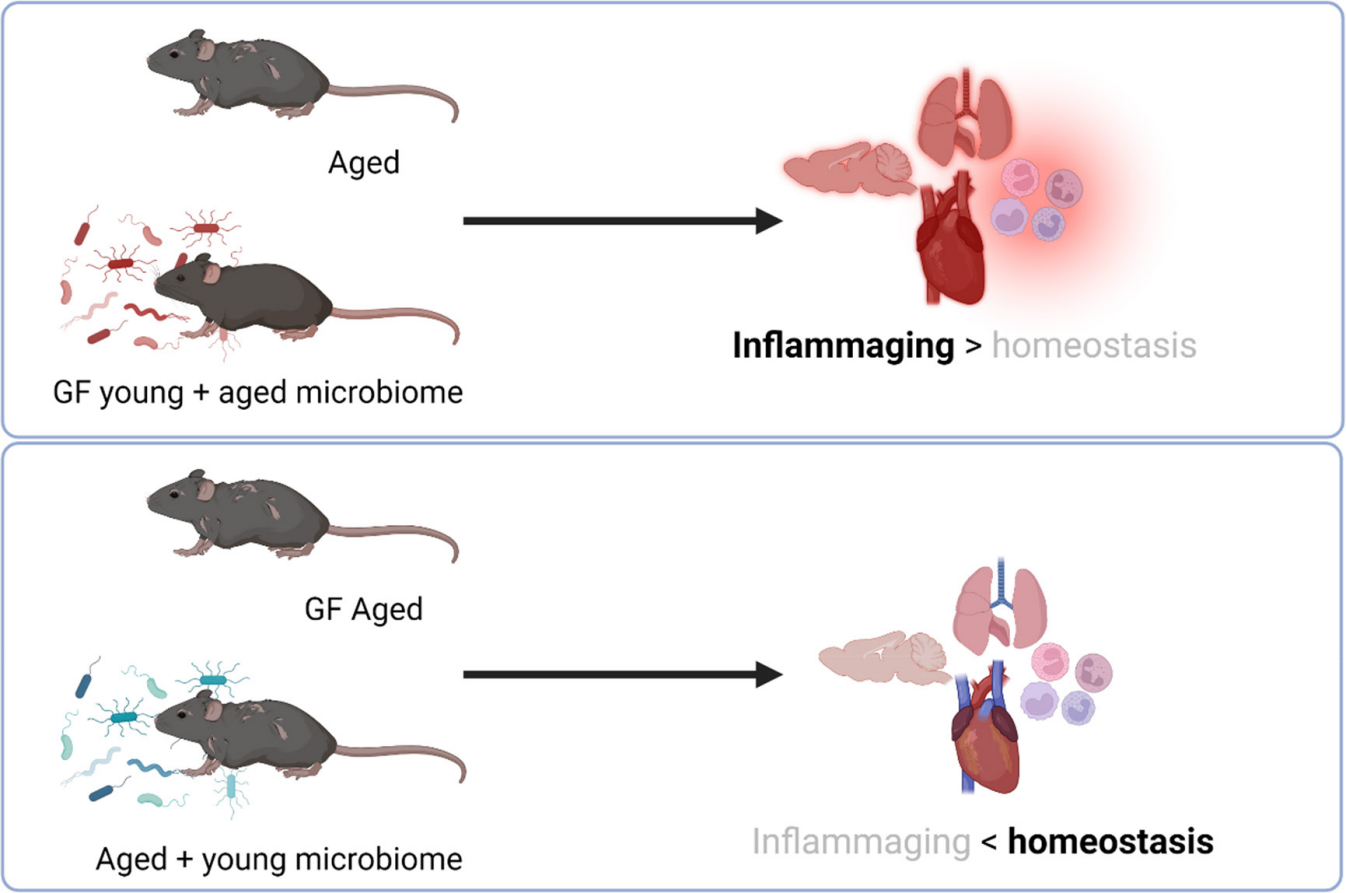
Impact of the age of the microbiota on inflammaging outcomes. Transfer of an aged microbiota is sufficient to elicit aspects of an inflammaging phenotype in young mice, including increases in circulating inflammatory cytokines and impaired phagocytosis. Conversely, aged germ‐free (GF) mice are resistant to inflammaging, and transfer of young microbiomes can reverse some aspects of immune homeostasis in conventionally raised aged mice

Supporting the hypothesis that the microbiota is a key driver of inflammaging and immune dysregulation, GF mice have a longer lifespan than their conventional (CNV) counterparts. In contrast to age‐matched CNV mice, GF mice exhibited no detectable increase in circulating IL‐6 (a hallmark of inflammaging) and maintained intestinal barrier integrity as well as macrophage antimicrobial activity (Thevaranjan et al., 2017). Further, microbial transfer via co‐housing with aged mice conferred age‐related inflammatory phenotypes (including increased levels of circulating TNFα and intestinal permeability) to young and old GF recipients (Thevaranjan et al., 2017). Notably, these phenotypes were not replicated by co‐housing with young mice, suggesting that the age of the microbiota (not just microbial exposure) matters (Figure 2). An independent study using fecal microbiota transplant (FMT) of young vs aged donors into young GF recipients demonstrated that an “aged” gut microbiota was uniquely capable of eliciting small intestinal inflammation (Figure 2), elevated levels of circulating microbiota‐derived inflammatory components, and systemic increases in T cell activation (Fransen et al., 2017). Notably, in a reciprocal experiment, FMT from young donors to aged recipients antagonized certain age‐related immune alterations in the mesenteric lymph nodes, including accumulation of migratory CD103^+^ DCs and recently activated CD8^+^ T cells (Boehme et al., 2021). For the purposes of experimental replication, it is important to note that in this particular study, young and aged mice were acquired from different commercial vendors and recipient mice retained an intact microbiota prior to FMT (Boehme et al., 2021).

Whether an aged microbiota directly contributes to impaired protective immunity, or whether microbiome manipulation can restore efficacy in aged mice, remains minimally examined. However, in humans, there is an association between increased age and more severe clinical outcomes following *C. difficile* infection (Henrich et al., 2009; Pépin et al., 2005). Further, in a study examining *C. difficile* in young and aged mice, microbiota exchange resulted in a significantly improved early immune response and survival in aged mice, providing support for the modulation of the aged microbiome as a method of modifying protective immunity, although the requisite pretreatment with antibiotics in order to establish productive *C. difficile* infection in immunocompetent mice slightly confounds the interpretation of this study (Shin et al., 2018).

Emerging data indicate that an aged microbiota can influence immune cells at distal sites, including CNS‐resident microglia (Boehme et al., 2021; D'Amato et al., 2020; Golomb et al., 2020). The observation that microglia in GF mice have a less activated phenotype than conventionally housed mice harboring a complex microbiota, and that a “normal” activation state could be restored by SCFA supplementation (Erny et al., 2015), was critical to developing a framework for investigation of gut–brain communication. The microbiota‐dependent effects on activation, morphology, and function of microglia have been associated with cognitive and behavioral effects, and their inflammatory and debris‐clearing functions are being explored as contributors to age‐related neurodegeneration (Lee et al., 2020; Mossad & Blank, 2021).

Collectively, these studies in mouse models and humans suggest that aging has the potential to drive significant changes to the intestinal microbiota and its associated metabolites, as well as localized intestinal and systemic immune responses. However, the relative contribution of environmental influences vs cell‐intrinsic functional changes in the development of age‐associated dysbiosis, immunosenescence, and inflammaging remains incompletely understood. Given that aged GF mice are protected against age‐associated inflammation (Thevaranjan et al., 2017), and an aged FMT is sufficient to confer inflammaging (Fransen et al., 2017; Thevaranjan et al., 2017), which can potentially be reversed by young FMT (Boehme et al., 2021; Figure 2), at least some of these changes appear to be microbiota‐influenced. Mechanistic understanding of how the microbiota influences immune homeostasis and function throughout the aging process is critical. In addition to direct host–microbe interactions, including those caused by increased translocation of bacteria and bacterial products through the gut mucosa with age, accumulating data indicate that differences in the intestinal microbiome correlate with healthy vs unhealthy aging (O'Toole & Jeffery, 2015; Parker et al., 2022; Ragonnaud & Biragyn, 2021; Thevaranjan et al., 2017).

The microbiota and microbiota‐derived metabolites impact IEC activity and composition, which assist in relaying aberrations in intestinal homeostasis to the broader immune system (Ornelas et al., 2022; Peterson & Artis, 2014). The microbiota, its metabolites, and IECs undergo a host of changes during the aging process. The interplay between these factors throughout aging and their impact on the progression of inflammaging and immunosenescence remains an understudied and important area of inquiry.

## 3 INTESTINAL EPITHELIAL CELL POPULATIONS DURING THE AGING PROCESS

**FIGURE 3:**
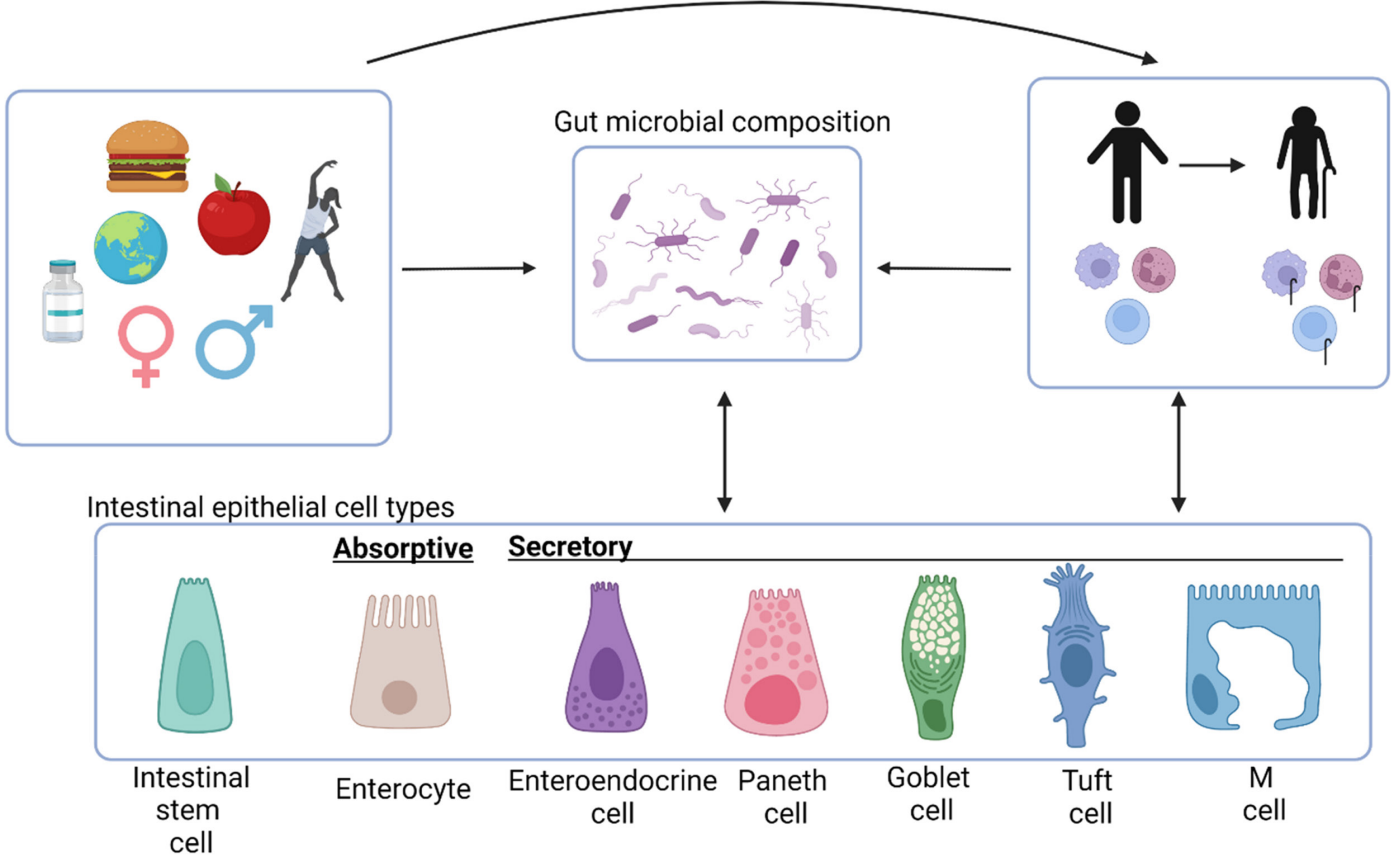
Intestinal epithelial cells at the intersection of environmental factors, the microbiota, and immunity. During the aging process, immune cells, gut microbial composition, and intestinal epithelial cells are each impacted by a multitude of factors including environmental factors (geography, immunological exposure, infection history, medical history (type of birth, antibiotic use, etc)), age, sex, and genetics. The tripartite communication between these systems could be manipulated to promote healthy aging outcomes

The intestinal epithelium is populated by a diverse group of differentiated IECs derived from intestinal epithelial stem cell (iESC) precursors, which serve a variety of distinct functions to maintain intestinal homeostasis, including communication with the gut microbial community. These cells include absorptive (colonocytes in the large intestine and enterocytes in the small intestine) and secretory (goblet cells, Paneth cells, enteroendocrine cells, M cells, and tuft cells) cell types (Clevers, 2013), which function as critical conveyers of environmental and microbiota signals to the host immune system (Figure 3).

Lgr5^+^ iESCs in intestinal crypts are the progenitors of all IEC subtypes (Barker et al., 2007). Both human and murine IECs express a variety of toll‐like receptors (TLRs), including TLR1, TLR2, TLR4, TLR5, and TLR9 (Abreu, 2010). T helper cytokines have a dramatic impact on IEC function and rejuvenation. Tregs produce IL‐10, which promotes iESC replenishment of the intestinal epithelium, while Th1‐derived interferon (IFN)‐γ drives expression of MHCII on iESCs (Biton et al., 2018), allowing them to function as unconventional antigen‐presenting cells (APCs). Intestinal microbial populations, IECs, and immune signaling undergo dramatic changes during the aging process (Bosco & Noti, 2021; Müller et al., 2019; Nicoletti, 2015; Walrath et al., 2021); however, the implications of these changes on the complex interplay between these systems remain minimally understood. Here, we review available literature on epithelial barrier function and IEC subtypes in the context of aging, highlighting areas where these relationships remain unclear, and in other cases suggesting hypotheses to link IECs to microbiota and/or immune function. This topic was also recently reviewed in the broader context of the interplay of these factors on other organs (Walrath et al., 2021).

### 3.1 Age‐associated alterations to intestinal barrier function

The mucus layer is located between the microbiota and IECs and is composed of highly glycosylated mucins. This layer provides a physical, dividing interface and protection against digestive enzymes and pathogenic microbes in addition to acting as a nutritional source for anaerobic members of the microbiota (Ali et al., 2020). Goblet cells are primarily responsible for the secretion and renewal of the mucus layer through the production of Muc2, a highly *O*‐glycosylated protein. Defective mucus production is associated with the development of chronic inflammation as seen in spontaneous colitis (Heazlewood et al., 2008; Turner, 2009). During the aging process, the gut mucus layer can go through dramatic alterations, including a six‐fold reduction in the thickness of the colonic mucus layer in old vs young littermate mice that leads to more frequent direct IEC–microbiota interactions (Sovran et al., 2019). Interestingly, male mice are more susceptible to age‐associated decreases in colonic mucus thickness than female mice (Elderman et al., 2017). Studies employing aged mice have documented microbiota‐dependent defects in intestinal barrier integrity that in conventionally housed mice with complete microbiomes were associated with altered β‐diversity and increased bacterial product translocation relative to young controls (Binyamin et al., 2020; Boehme et al., 2021; Conley et al., 2016; Parker et al., 2022; Sovran et al., 2019; Thevaranjan et al., 2017). Supplementation with both *Lactobacillus plantarum* WCFS1 (van Beek et al., 2016) and *Akkermansia muciniphila (*van der Lugt et al., 2019 *)* has been suggested as potential probiotic strains to protect against age‐related decline of the mucus barrier and was found to protect mucus barrier integrity in the *Ercc1* ^*−/Δ7*^ murine model of accelerated aging.

IECs themselves provide a second layer of physical separation between the microbiota and luminal contents and the surrounding tissues. This barrier effect is mediated by tight junction proteins between IECs such as occludins (Furuse et al., 1993), claudins (Furuse, Fujita, et al., 1998; Furuse, Sasaki, et al., 1998), and zonula occludens (ZO‐1, ZO‐2) (Stevenson et al., 1986; Umeda et al., 2006). In colonic tissue from old baboons, tight junction protein expression is reduced, resulting in impaired barrier integrity and increased permeability of colonic biopsies in addition to elevated production of inflammatory cytokines (Tran & Greenwood‐Van Meerveld, 2013). Gut microbial composition has been shown to correlate with circulating inflammatory cytokine levels and health in elderly nursing home patients (Claesson et al., 2012), rats (Li et al., 2020), and mice (Conley et al., 2016). However, there is conflicting evidence in humans as to whether aging results in increased gut permeability (Man et al., 2015; Wilms et al., 2020), despite the association of aging with increased levels of serum zonulin (Qi et al., 2017), a known regulator of intestinal permeability (Sturgeon & Fasano, 2016). While the impact of age on human intestinal barrier integrity remains less clear, altered epithelial barrier integrity allowing translocation of commensal microbes, microbial debris, and/or luminal metabolites represents a promising explanation for the aberrant inflammaging seen with advancing age (Akdis, 2021).

### 3.2 Intestinal epithelial stem cells

Lgr5^+^ iESCs in the intestinal crypts are the progenitors of all IEC subtypes (Barker et al., 2007), which may act as unconventional MHCII‐expressing APCs under inflammatory conditions and contribute to epithelial‐cell remodeling after the resolution of an infection (Biton et al., 2018). These cells asymmetrically divide as regulated by Notch signaling and give rise to two different daughter cells, a self‐replacing stem cell and a differentiating cell (Srinivasan et al., 2016). The differentiating daughter cell initially becomes a transit‐amplifying progenitor and undergoes another 4–5 rounds of division before differentiating into a mature IEC subtype. As these cells mature, absorptive IECs (colonocytes, enterocytes) go through programmed cell death and are sloughed off, allowing for renewal of the intestinal epithelium every 3–5 days (Cheng & Leblond, 1974). Appropriate regulation of iESC proliferation and IEC renewal is a critical aspect of intestinal homeostasis.

**FIGURE 4:**
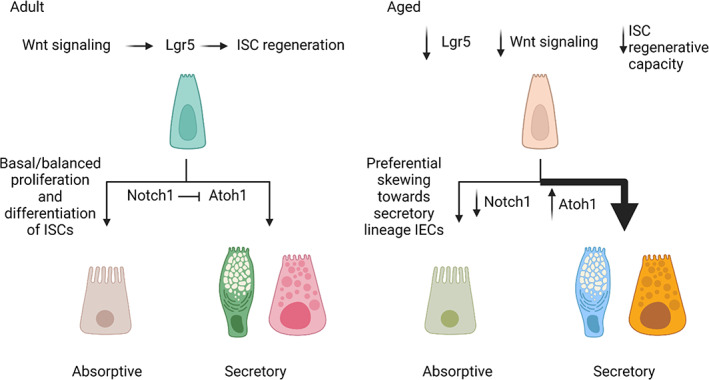
Changes in IEC differentiation during the aging process. Aging results in silencing of the stem cell marker *Lgr5*, resulting in a reduction in Wnt signaling and iESC regenerative capacity. *Notch1*‐mediated inhibition of *Atoh1* regulates the differentiation of secretory IECs and maintains balanced differentiation of IEC subtypes in healthy human adults. Decreased expression of *Notch1* with age results in a corresponding increase of *Atoh1* and preferential skewing toward secretory IEC fate

In aged individuals, iESCs become dysfunctional, resulting in a diminished capacity to regenerate the intestinal epithelium and maintain intestinal homeostasis. In alignment with these observations, iESCs and crypt cultures from aged mice exhibit reduced expansion and fewer, less complex enteroids devoid of differentiated cell types with reduced expression of stem cell markers (Olfm4, Bmi1, and Hopx) and cell‐type specific markers Alpi (enterocytes), Defa24 (Paneth), and Chga (enteroendocrine cells), but not Atoh1 (secretory lineage) (Cui et al., 2019; Moorefield et al., 2017). IEC organoids from aged mice exhibit epigenetic silencing of the stem cell marker *Lgr5*, resulting in a reduction of Wnt signaling and cell proliferation (Uchida et al., 2018). Aged iESCs also show a reduction in *Notch1* expression and a corresponding increase in *Atoh1* expression (Nalapareddy et al., 2017), an important component in the balance of iESC activity regulated by Wnt and Notch signaling (Tian et al., 2015). Notably, restoration of Wnt signaling can rescue the proliferative defects characteristic of aged iESCs (Nalapareddy et al., 2017). Collectively, these studies suggest an overall reduction in the capacity of aged iESCs to regenerate the intestinal epithelium, resulting in a diminished maintenance of intestinal integrity and homeostasis (Figure 4). As these experiments were performed with cells isolated from conventionally housed mice, it remains unknown whether this is an age‐intrinsic effect, or if an aging microbiota may contribute to functional impairments.

### 3.3 Enterocytes

Enterocytes are the most abundant IEC in the intestine and have a characteristic microvilli brush border on their apical surface. Given their abundance, enterocytes are critical for maintaining epithelial barrier integrity. Enterocytes are critical for the absorption and export of luminal nutrients and undergo age‐related changes in nutrient absorption, consistent with the known changes in metabolic pathways during the aging process (Barzilai et al., 2012; Yoshimoto et al., 2021). Enterocytes from elderly, otherwise healthy, people demonstrate increased rates of both apoptosis and proliferation, which may lead to reduced enterocyte maturity and absorptive efficacy (Ciccocioppo et al., 2002). Consistent with these observations, aged mice have cleaved caspase‐3 positive cells in their intestinal crypts and villi, suggesting increased rates of apoptosis (Moorefield et al., 2017). Further, enterocytes of aged rats expressed less enterocyte fatty acid–binding protein and had reduced intestinal lipid uptake (Woudstra et al., 2004), supporting age‐related alterations in nutrient absorption. Collectively, these data suggest that changes in IEC nutrient absorption may contribute to the alterations in microbiota composition, microbiota‐derived metabolites, and downstream immune signaling seen in aging populations.

While the primary function of these absorptive cells is to absorb and export luminal nutrients, enterocytes also produce antimicrobial proteins (AMPs), including β‐defensin and the secreted C‐type lectin regenerating islet‐derived protein IIIγ (REGIIIγ). REGIIIγ, which is derived from both enterocytes and Paneth cells, is essential for enforcing the so‐called “microbial firewall” (Hooper & Macpherson, 2010), as evidenced by the increased microbial colonization of the gut epithelium and corresponding increase in local Immunoglobulin A (IgA) and IFNγ in REGIIIγ^−/−^ mice (Vaishnava et al., 2011). The epithelial‐derived alarmin cytokine IL‐33 is a critical primer for Th2 and Treg cell differentiation (Duan et al., 2012), but is also essential for optimal REGIIIγ expression. In the absence of IL‐33, REGIIIγ expression is reduced, resulting in overgrowth of the colonic microbiota with an altered community profile (Xiao et al., 2019). Given the importance of REGIIIγ for the maintenance of intestinal epithelium integrity, REGIIIγ and IL‐33 may represent interesting targets to further investigate in the context of aging. Consistent with this, REGIIIγ is elevated in the ileum of aged mice (Tremblay et al., 2017), though there has been little study of the human homolog REG3A in the intestinal epithelium. It is tempting to speculate that shifts in the microbial community, diminished mucus thickness, and the increased frequency of microbe–epithelial contact that are associated with aging may result in dynamic regulation of intestinal REGIIIγ expression.

### 3.4 Goblet cells

Goblet cells comprise 10%–15% of the intestinal epithelium (17% of epithelial cells in the colon, and 5% of epithelial cells in the small intestine (Nyström et al., 2021)), making them the most abundant secretory IEC. A central function of goblet cells is the production and secretion of the highly *O*‐glycosylated protein Muc2, the primary component of the mucus layer. While goblet cell production of mucins is a key function, goblet cell‐derived trefoil factor 3 (TFF3) and resistin‐like molecule‐β (RELMβ) also contribute to intestinal barrier integrity. TFF3 promotes mucin cross‐linking and viscosity, as well as IEC resistance to apoptosis, and migration during wound repair (Dignass et al., 1994; Taupin et al., 2000; Thim et al., 2002). RELMβ promotes secretion of Muc2 (Krimi et al., 2008), regulates macrophage and CD4^+^ T cell responses in response to helminth infection (Nair et al., 2008), and promotes CD4^+^ T cell recruitment and mediation of IEC hyperplasia during pathogenic intestinal bacterial infection (Bergstrom et al., 2015).

GF mice have dramatically reduced levels of Muc2 compared with conventional mice, resulting in a thin mucus layer (Bergström et al., 2012) reminiscent of the mucus barrier reduction seen in aged mice (Sovran et al., 2019). When exposed to a conventional microbiota, GF mice produce significantly more TFF3 and RELMβ, resulting in a thickened mucus barrier (He et al., 2003). These observations are consistent with the effect of age‐related dysbiosis on mucus integrity and suggest that a healthy, “young” gut microbiota contributes to immune homeostasis through maintenance of the mucus barrier.

Despite a skewing of iESCs toward the secretory lineage with aging (Nalapareddy et al., 2017), literature findings on goblet cells in the aged epithelium are mixed, with conflicting reports of both increased and decreased goblet cell numbers in the small intestine during murine aging (Nalapareddy et al., 2017; Powell et al., 2020; Sovran et al., 2019; Tremblay et al., 2017). Interestingly, one study found that not only are goblet cells present in higher amounts in the villi of aged mice, but they contain larger mucin granules that stain more intensely with the mucin stain Alician Blue, suggestive of impaired mucin secretion by aged goblet cells (Tremblay et al., 2017). Examination of goblet cells in the colon of aged mice found that while goblet cell number was not affected, aged goblet cells exhibited higher levels of caspase‐3 expression and increased apoptosis (Sovran et al., 2019). While the differential findings in the literature make accurate interpretation of these studies challenging, collectively these findings suggest that while goblet cell numbers may not be affected by age, changes in goblet cell functionality may contribute to compromised barrier integrity.

In addition to altered goblet cell functionality, aged mice also exhibit changes to the intestinal microbiota (Elderman et al., 2017). Colonization of geriatric mice with indole‐secreting bacteria has been shown to increase IEC proliferation and promote goblet cell differentiation (Powell et al., 2020). Given that GF mice also show significant defects in mucus thickness (Bergström et al., 2012), these findings suggest a strong link between goblet cell function/mucus production with the microbiota and age, which likely influences immune homeostasis.

### 3.5 Paneth cells

Paneth cells are a specialized subset of secretory IECs found in small intestinal crypts of Lieberkühn, with approximately 5–15 Paneth cells per crypt (Bry et al., 1994). These cells are packed with antimicrobial peptides and dense granule proteins, including defensins, cathelicidins, and lysozyme that can migrate to the mucus layer, embedding antimicrobial activity into the physical protective barrier (Meyer‐Hoffert et al., 2008; Vaishnava et al., 2008). Paneth cells contribute to homeostasis at the host–microbiota interface via TLR‐dependent recognition of microbe‐associated molecular patterns (MAMPs) expressed by the gut commensal microbiota, activation of MyD88, and secretion of AMPs that impact microbial community composition and limit bacterial permeation into tissues (Vaishnava et al., 2008). In particular, an elegant study using mice that either expressed human α‐defensin gene (h*DEFA5*) or lacked α‐defensin function through genetic ablation of the α‐defensin processing enzyme matrix metalloproteinase 7 (MMP7) demonstrated that Paneth‐cell‐derived α‐defensin directly shapes the microbiota. h*DEFA5*‐expressing mice exhibited reduced Segmented Filamentous Bacteria (SFB) with a population shift toward *Bacteroides*, while *Mmp7* ^*−/−*^ mice showed an increase in Firmicutes, with a reciprocal decrease in *Bacteroides*, supporting a direct role for Paneth‐cell‐derived α‐defensin in shaping the microbiota (Salzman et al., 2010). All experimental litters in this study were bred and maintained in the same SPF animal facility, suggesting that differences from WT controls were due to the experimental parameters and not institutional variability.

In aged mice, expression of the Paneth‐cell‐derived AMPs lysozyme (Sovran et al., 2019) and α‐defensin (Tremblay et al., 2017) is reduced compared with young littermate controls, which was associated with compromised barrier integrity. Conflicting data between these two studies regarding the impact of age on the expression of the AMP angiogenin 4 (Ang4) suggest that this particular AMP may be more significantly impacted by facility variations in the microbiota than by age (Sovran et al., 2019; Tremblay et al., 2017). Overall, studies of the age‐associated changes to Paneth cells remain minimal and the potential for these changes to impact the microbiota composition, resistance to intestinal bacterial colonization and translocation, and inflammaging merits further investigation.

### 3.6 Enteroendocrine cells

Enteroendocrine cells (EECs) make up ~1% of the gut epithelium and span the entire gastrointestinal tract. EEC sensing of luminal nutrients elicits secretion of peptide hormones to mediate digestion (Sternini et al., 2008), immunity (Li et al., 2011; Zhang et al., 2011, 2014), and neuronal signaling (Rhee et al., 2009). As with other IECs, EECs develop from Lgr5^+^ iESC precursors, and their differentiation is facilitated by coordinated expression of the transcription factors Atoh1, neurogenin3, and neurogenic differentiation 1 (NEUROD1). EECs can be specifically identified by the expression of the surface marker Claudin‐4 (Nagatake et al., 2014), which is present on all EEC subtypes. Until recently, EEC subtypes were thought to be characterized by secretion of unique hallmark peptide hormones (Worthington et al., 2018), though more recent work using transgenic mouse models suggests a more flexible and less terminally differentiated EEC secretome, with co‐secretion of a given peptide hormone being most closely linked to the tissue environment (Adriaenssens et al., 2015; Egerod et al., 2012; Grunddal et al., 2016; Habib et al., 2018; Svendsen et al., 2015). EECs and their associated peptide hormones, which may exert a variety of effects on immune, neuronal, and metabolic activity, were recently and thoroughly reviewed by Worthington et al. (Worthington et al., 2018).

Aged mice exhibit increased numbers and activity of K cells, an EEC subtype that produces glucose‐dependent insulinotropic polypeptide/gastric inhibitory polypeptide (GIP), which functions in a positive feedback loop: K‐cell‐derived GIP secretion is upregulated by and promotes the accumulation of fat. K cell hyperplasia‐associated GIP hypersecretion promotes the build up of fat and insulin resistance in aged mice, which was ameliorated by ablation of the GIP receptor (Ikeguchi et al., 2018) or GIP itself in aged mice (Kanemaru et al., 2020). Consistent with this, older humans also exhibit increased circulating levels of GIP following glucose consumption (de Jesús Garduno‐Garcia et al., 2018), suggesting that increased GIP is conserved across species and that the amelioration of age‐associated fat and inflammation buildup seen in mice following GIP manipulation may be reproducible in humans. Older humans also have increased numbers of enterochromaffin cells (Yu et al., 2016), which secrete the hallmark hormone serotonin. Altered levels of and signaling by serotonin play a role in a variety of neurodegenerative disorders including Parkinson's disease (Fox et al., 2009; Politis & Niccolini, 2015), multiple sclerosis (Malinova et al., 2018; San Hernandez et al., 2020), and Alzheimer's disease (Mdawar et al., 2020; Smith et al., 2017). The impact of aging on enteroendocrine cells remains understudied but given the role of this IEC subtype in the sensing of and response to luminal contents and the microbiota, and their systemic effects, these cells may represent major players in the regulation of aging process and age‐associated comorbidities.

### 3.7 M cells

M cells, or microfold cells, differentiate from iESCs in a receptor activator of nuclear factor‐κB ligand (RANKL)‐ and Spi‐B‐dependent manner and mediate nonspecific sampling of luminal antigens and intact microorganisms. APCs then migrate to small intestinal Peyer's Patches (PPs), organized lymphoid structures containing T cells, B cells, and DCs embedded below the epithelium of the small intestine, where they stimulate IgA production through induction of a germinal center (GC) reaction. Antigen sampling by M cells is critical for the production of enteric commensal reactive IgA (Rios et al., 2016) by intestinal plasma cells. Commensal specific IgA is then secreted to the intestinal lumen where it contributes to microbiota tolerance, another key component of the host‐derived firewall against microbial dysbiosis, and community organization (Bunker & Bendelac, 2018; Macpherson et al., 2018; Macpherson & Uhr, 2004). Consistent with this, IgA‐deficient humans exhibit an altered gut microbial composition compared with healthy controls (Catanzaro et al., 2019), which is consistent with the increased relative abundance of Proteobacteria and intestinal inflammation seen in IgA‐deficient mice (Mirpuri et al., 2014).

At steady state, M cells represent ~10% of the IECs in the follicle‐associated epithelium (FAE), which covers the luminal side of the lymphoid follicles of the gut‐associated lymphoid tissue (GALT) such as PPs (Mabbott et al., 2013). As mice age, mature M cell number in the FAE of PPs declines, resulting in reduced uptake and transcytosis of luminal antigens and microorganisms. This decline in the numbers of mature M cells is associated with decreased expression of CCL20 in the FAE, resulting in reduced B cell recruitment (Kobayashi et al., 2013).

Mouse studies support an age‐associated decline of IgA in the intestinal lumen (Koga et al., 2000), coincident with a reduction in mature M cells. Notably, this attrition of mature M cells can be reversed via exposure of aged mice to a young mouse microbiota, suggesting that the age‐related loss of mature M cells is linked to the microbiota (Donaldson et al., 2020). Somewhat counterintuitively, but consistent with an elevated ratio of memory: naive B cells in older individuals (Agarwal & Busse, 2010), increased levels of serum IgA are associated with older age in humans (Arranz et al., 1992; Khan et al., 2021). This conflicting finding between murine and human systems may be due to inherent mouse vs human differences, or aging may be associated with local IgA reductions in the intestinal lumen, but not systemic serum changes in IgA. While there is some conflicting evidence between mouse and human systems that needs to be untangled, age‐associated changes in the persistence and maturation of M cells may have a considerable impact on the regulation of intestinal homeostasis and immune‐mediated containment of luminal contents and the microbiota.

### 3.8 Tuft cells

Tuft cells are rare, secretory epithelial cells that can be identified by their unique expression of doublecortin and calcium/calmodulin‐dependent protein kinase‐like‐1 (DCLK1, also known as DCAMKL‐1; Gerbe et al., 2009) and the calcium‐activated ion transient channel receptor potential cation channel subfamily M member 5 (TRPM5). Although tuft cell chemosensation has been associated with protective immunity in the respiratory tract, their function in the intestine was more mysterious until a series of papers in 2016 demonstrated their vital role in activating type 2 immune responses to helminth and protozoa (Gerbe et al., 2016; Howitt et al., 2016; Schneider, 2021; von Moltke et al., 2016). Since then, these chemosensory cells have been hypothesized to act as immune sentinels, which has been confirmed by recent demonstrations that tuft cells can “sense” metabolites produced by bacterial and protozoal members of the microbiota and helminths through a succinate receptor (SUCNR1) (Banerjee et al., 2020; Nadjsombati et al., 2018) and Tas2r bitter‐taste receptors (Luo et al., 2019). The presence of tuft cells in GF mice indicates that their development is not microbiota‐dependent (McKinley et al., 2017).

Another function of DCLK1^+^ tuft cells is regulating IEC survival and self‐renewal responses following radiation‐induced DNA damage, as evidenced by increased apoptosis and hypoplasia in tuft‐cell‐deficient mice (Chandrakesan et al., 2016). DNA damage accumulates in human hematopoietic stem cells (HSCs) during aging (Rübe et al., 2011), thus while tuft cell function in aging remains understudied, we propose that diminished tuft cells may factor into the loss of IEC maintenance and proliferative capacity during the aging process. Further, considering the importance of tuft cells for epithelial renewal following radiation‐induced DNA damage in the context of thymic tuft cells, which play a role in shaping thymocyte development (Miller et al., 2018), there may be interesting implications for the role tuft cells play in both stunted rejuvenation of the intestinal epithelium and the thymic involution individuals undergo during the aging process.

Overall, IECs are critical for barrier function and maintaining immune homeostasis in the intestine. This is already well demonstrated in young individuals with intestinal inflammation, such as IBD patients who present with microbial dysbiosis, IEC dysregulation, and defective barrier integrity (Martini et al., 2017; Nishida et al., 2018). During the aging process, IEC populations, the microbiota they interface with, and the systemic host immune state undergo dramatic changes. Better understanding of the interplay between these populations during the aging process and inflammaging represents a critical area of research to better understand potential factors impacting age‐associated comorbidities.

## 4 AGE‐RELATED IMMUNE ALTERATIONS AND THE MICROBIOTA

An intact microbiota supports the generation of optimal protective immune responses to multiple infections in mice (Hagan et al., 2019; Ichinohe et al., 2011; Lynn et al., 2021). This finding has also been validated in healthy humans: following vaccination or infection with H1N1 influenza, antibiotic‐treated individuals with low preexisting antibody titers exhibit dampened induction of antigen‐specific IgG1 and IgA antibody responses, suggesting that priming of a novel immune response is compromised (Hagan et al., 2019). Collectively, these data suggest that antibiotic‐induced dysbiosis and impaired generation of appropriate adaptive immune responses are conserved across species. In the context of age‐related dysbiosis, this suggests that modulation of the aged microbiota may represent an appealing avenue to enhance protective immunity and vaccination responses in elderly individuals (Lynn et al., 2021; Lynn & Pulendran, 2018).

PPs represent a key location at which signals from the intestinal lumen and IECs are translated into an immune response via the production of cytokines and antibodies. The organized lymphoid architecture of PPs is central to T‐cell‐dependent IgA class‐switch recombination, as PPs are the site of GC reactions that support B cell receptor somatic hypermutation and affinity maturation of IgA class‐switched B cells. In contrast to the constitutive formation of GCs in the PPs in response to M‐cell‐derived microorganisms and luminal antigens in healthy adult mice, aged mice have defective GC reactions in PPs and have reduced activation and numbers of conventional CD11c^+^ DCs in PPs (Kato et al., 2003; Reboldi & Cyster, 2016; Stebegg et al., 2019). Consistent with this, aged mice exhibit immunosenescence via reduced antigen‐specific T cell proliferation, effector cytokines, and antibody production at both systemic (spleen) and mucosal (PP) sites compared with young controls (Koga et al., 2000) as well as diminished oral tolerance as a consequence of PP dysfunction (Kato et al., 2003). FMT from young mice into older recipients can restore defective GC reactions in PPs, although the restorative effect did not extend to peripheral lymph nodes, suggesting a localized effect (Stebegg et al., 2019). Collectively, these findings suggest that some of the disruption in protective and tolerant mucosal immune responses associated with age may be due to altered PP function and that these defects are intimately tied to the aging microbiota.

In addition to adaptive immune alterations, increased age is associated with dramatic changes to the innate immune response and altered activity of HSCs, neutrophils, monocytes, macrophages, natural killer (NK) cells, and myeloid‐derived and plasmacytoid dendritic cells in both human and mouse models. While the majority of these cell populations exhibit age‐related functional changes, the bulk numbers of innate cell populations remain largely unchanged in aged individuals (Shaw et al., 2013).

Aged mice and humans exhibit an increased propensity for HSC skewing toward myeloid lineage differentiation over lymphopoiesis (Cho et al., 2008; Pang et al., 2011; Rossi et al., 2005), which is consistent with the reduction in naive T cell numbers and T cell receptor diversity seen in older individuals (Appay & Sauce, 2014). Providing evidence that the microbiota may contribute to this characteristic skewing of hematopoiesis, HSC isolated from aged conventional mice demonstrates the typical elevation in myeloid: lymphoid progeny, while the developmental potential of HSC isolated from aged GF mice was similar to that of young, conventional mice. Data from IL‐1Rα‐deficient mice suggest a potential mechanistic link. In the absence of IL‐1Rα, mice acquire a dysbiotic microbiome that is associated with Th17 cell expansion in the small intestinal lamina propria. This phenotype was microbiota‐dependent and transferable via FMT, demonstrating that the microbiota of *il1ra* ^−/−^ mice is both necessary and sufficient to promote intestinal Th17 differentiation (Rogier et al., 2017). Interestingly, aged specific‐pathogen free (SPF) mice produce elevated levels of IL‐1α/β that could be diminished via antibiotic‐mediated depletion of the microbiota (Kovtonyuk et al., 2021). Further, genetic loss or pharmacologic blockade of IL‐1 signaling protects HSCs from developing age‐associated inflammatory signatures and restores the unbiased lymphoid‐myeloid differentiation seen in young HSCs (Kovtonyuk et al., 2021). Collectively, these data suggest that changes in the aged microbiota are detected in the bone marrow and impact HSC function with downstream effects on immune homeostasis.

Neutrophils are rapid, front‐line responders of the innate immune response. Aging results in altered neutrophil bone marrow egress, migration, and chemotaxis (Martin et al., 2003; Niwa et al., 1989; Nomellini et al., 2012), as well as reduced phagocytic and bactericidal activity (Wenisch et al., 2000). Further, elderly individuals also exhibit higher rates of spontaneous neutrophil reactive oxygen species (ROS) production (Ogawa et al., 2008), which may contribute to the inflammaging process (Liguori et al., 2018). The microbiota is a major mediator of neutrophil aging, and antibiotic‐mediated depletion of the microbiota in aged mice diminishes the number of circulating neutrophils and neutrophil‐attributed inflammation (Zhang et al., 2015).

Overall, aged macrophages are less responsive to infection and exhibit reduced cytokine production (Renshaw et al., 2002; Shaik‐Dasthagirisaheb et al., 2010), phagocytosis, and TLR expression/signaling (Boehmer et al., 2005; Liang et al., 2009; Renshaw et al., 2002), as well as increased expression of markers of senescence (Hall et al., 2016). Age‐related microbial dysbiosis does not result in differential expression of maturation markers on macrophages (CD11b, Ly6C, F4/80, and Ly6G); however, aged macrophages are significantly less capable of conducting bacterial killing compared with young macrophages and aged GF mouse‐derived macrophages better maintain their antimicrobial activity compared with their conventional counterparts (Thevaranjan et al., 2017). Monocytes are macrophage precursors that also undergo age‐associated functional alterations, including reduced efferocytosis (de Maeyer et al., 2020), phagocytosis (Hearps et al., 2012), type I IFN production (Molony et al., 2017), and TLR signaling (Nyugen et al., 2010; van Duin et al., 2007). Strikingly, age‐related microbial dysbiosis, and more specifically, age‐related reduction in the commensal bacterium *Akkermansia muciniphila*, which metabolizes fibers into SCFAs such as acetate, propionate, and butyrate, results in not only impaired barrier integrity and leakiness, leading to increased bacterial products in the circulation, but a corresponding increase in the activation of CCR2^+^ inflammatory monocytes (Bodogai et al., 2018).

Notably, despite increased skewing of HSCs toward the myeloid lineage, the number of circulating myeloid DCs progressively declines with age (Della Bella et al., 2007; Orsini et al., 2012). Aged DCs have a reduced capacity to macropinocytose and endocytose antigens (Agrawal et al., 2007), initiate T cell activation (Moretto et al., 2008), and migrate in response to the chemokines CCL19 and CXCL12 (Agrawal et al., 2007). These phenotypes are reminiscent of the reduced numbers of intestinal DCs seen in GF mice (Chung et al., 2012; Walton et al., 2006), suggesting that the microbiota influences DC hematopoiesis, migration, or tissue residency, although the specific impact of the aged microbiome on DC function requires further examination.

This constellation of age‐associated immune changes, especially when considered in the context of IECs and the microbiota, provides a complex picture of potential aberrations, which may result in age‐associated comorbidities. Centenarians represent a unique subset of aged individuals who, while still undergoing substantial changes to both immune and microbial populations, exhibit improved longevity and quality of life while appearing to avoid the worst of inflammaging.

### 4.1 Inflammaging in centenarians

Aging does not occur equally across a population and untangling the unique attributes of successful agers has the potential to open the door to novel therapeutic strategies and interventions. Compared with seniors who exhibit a pro‐inflammatory inflammaging phenotype, centenarians, despite showing elevated levels of certain inflammatory parameters, also exhibit an antagonistic anti‐inflammatory immune response that is associated with longevity, referred to as anti‐inflammaging (Biagi et al., 2010). Consistent with this, a lower inflammation score (which considered circulating IL‐6, TNF‐α, and C reactive protein) (Giovannini et al., 2011) has been established as a strong predictor of all‐cause mortality (Giovannini et al., 2011; Marcos‐Pérez et al., 2018) and is associated with the longevity phenotype seen in centenarians (Arai et al., 2015). On the contrary, elevated levels of circulating IL‐6 (Biagi et al., 2010), IL‐22 (Basile et al., 2012), IL‐15 (Gangemi et al., 2005), and IL‐18 (Gangemi et al., 2003) have been detected in centenarians, which may contribute to improved infectious immunity and survival. Despite conflicting evidence on the IL‐6 levels in centenarians, suggesting that this inflammatory marker may vary based on the given population studied, a reduction in circulating TNFα, a known risk factor for frailty (Bruunsgaard et al., 2003; Van Epps et al., 2016) relative to other seniors, is a common theme among centenarians (Arai et al., 2015; Biagi et al., 2010). Considered together, these observations suggest a balancing of pro‐ and anti‐inflammatory immunity in centenarians, which contributes to successful aging.

The separate observation of a unique, highly diverse microbiota in centenarian “super‐agers” has yet to be mechanistically linked to these immune phenotypes but may be due to altered metabolite production. Diet and exercise, among other lifestyle factors, have a significant impact on successful aging and the composition of the microbiota of both young and elderly individuals (Claesson et al., 2012; David et al., 2014; Gopinath et al., 2018; Ramos et al., 2022; Sepp et al., 2022). However, the critical components for successful aging and the acquisition of healthy centenarian status remain elusive, as the markers of successful aging are complicated and multifactorial, with genetic, environmental, and immune factors all contributing to a state of homeostasis and improved quality of life (Motta et al., 2007). How IECs differ between centenarians and aged individuals who are younger but frailer remains understudied but may provide further clues to improved longevity.

## 5 CONCLUSIONS AND IMPLICATIONS

The complex interplay between the microbiota, IECs, and the broader immune system during the aging process represents an area of increasingly important study given the rising age of the global population and the resultant increases in common comorbidities, including cancer, metabolic, inflammatory, and neurodegenerative conditions.

IECs occupy a unique position as an interface between the microbiota and the immune system. We suggest that accumulating dysfunction of IECs with age likely contributes to the characteristic changes in microbial ecology, potentially creating novel nutritional niches as a result of altered enterocyte absorption or secretory cell function. A thinner mucosal barrier or reduced intestinal barrier integrity facilitates enhanced translocation of whole microbes, MAMPs, or metabolites into the circulation and/or other tissues, thereby contributing to inflammaging and/or immunosenescence. Thus, further studies that examine and quantify the phenotype and function of IECs in aged populations, long‐lived healthy centenarians, and those living with age‐related inflammatory conditions represent critical future research. Further investigation in this field may illuminate new therapeutic strategies, such as dietary interventions or supplementation with particular microbial community members, which could be used to improve both quality and expectancy of life for the aging global population.

## AUTHOR CONTRIBUTIONS

LSH wrote the manuscript and made figures with support and guidance from LCO.

## CONFLICT OF INTEREST

The authors have no conflicts to declare.

## References

[acel13700-bib-0001] Abreu, M. T. (2010). Toll‐like receptor signalling in the intestinal epithelium: How bacterial recognition shapes intestinal function. Nature Reviews Immunology, 10(2), 131–144. 10.1038/nri2707 20098461

[acel13700-bib-0002] Adriaenssens, A. , Lam, B. Y. , Billing, L. , Skeffington, K. , Sewing, S. , Reimann, F. , & Gribble, F. (2015). A transcriptome‐led exploration of molecular mechanisms regulating somatostatin‐producing D‐cells in the gastric epithelium. Endocrinology, 156(11), 3924–3936. 26241122 10.1210/en.2015-1301 PMC4606756

[acel13700-bib-0003] Agarwal, S. , & Busse, P. J. (2010). Innate and adaptive immunosenescence. Annals of Allergy, Asthma & Immunology: Official Publication of the American College of Allergy, Asthma, & Immunology, 104(3), 183–190; quiz 90–2, 210. 10.1016/j.anai.2009.11.009 20377107

[acel13700-bib-0004] Agrawal, A. , Agrawal, S. , Cao, J. N. , Su, H. , Osann, K. , & Gupta, S. (2007). Altered innate immune functioning of dendritic cells in elderly humans: A role of phosphoinositide 3‐kinase‐signaling pathway. Journal of Immunology, 178(11), 6912–6922. 10.4049/jimmunol.178.11.6912 17513740

[acel13700-bib-0005] Akdis, C. A. (2021). Does the epithelial barrier hypothesis explain the increase in allergy, autoimmunity and other chronic conditions? Nature Reviews Immunology, 21(11), 739–751. 10.1038/s41577-021-00538-7 33846604

[acel13700-bib-0006] Ali, A. , Tan, H. , & Kaiko, G. E. (2020). Role of the intestinal epithelium and its interaction with the microbiota in food allergy. Frontiers in Immunology, 11, 604054. 33365031 10.3389/fimmu.2020.604054 PMC7750388

[acel13700-bib-0007] Appay, V. , & Sauce, D. (2014). Naive T cells: The crux of cellular immune aging? Experimental Gerontology, 54, 90–93. 24440387 10.1016/j.exger.2014.01.003

[acel13700-bib-0008] Arai, Y. , Martin‐Ruiz, C. M. , Takayama, M. , Abe, Y. , Takebayashi, T. , Koyasu, S. , Suematsu, M. , Hirose, N. , & von Zglinicki, T. (2015). Inflammation, but not telomere length, predicts successful ageing at extreme old age: A longitudinal study of semi‐supercentenarians. eBioMedicine, 2(10), 1549–1558. 26629551 10.1016/j.ebiom.2015.07.029 PMC4634197

[acel13700-bib-0009] Arranz, E. , O'Mahony, S. , Barton, J. R. , & Ferguson, A. (1992). Immunosenescence and mucosal immunity: Significant effects of old age on secretory IgA concentrations and intraepithelial lymphocyte counts. Gut, 33(7), 882–886. 1644326 10.1136/gut.33.7.882 PMC1379398

[acel13700-bib-0010] Banerjee, A. , Herring, C. A. , Chen, B. , Kim, H. , Simmons, A. J. , Southard‐Smith, A. N. , Allaman, M. M. , White, J. R. , Macedonia, M. C. , Mckinley, E. T. , Ramirez‐Solano, M. A. , Scoville, E. A. , Liu, Q. , Wilson, K. T. , Coffey, R. J. , Washington, M. K. , Goettel, J. A. , & Lau, K. S. (2020). Succinate produced by intestinal microbes promotes specification of tuft cells to suppress ileal inflammation. Gastroenterology, 159(6), 2101–15.e5. 32828819 10.1053/j.gastro.2020.08.029 PMC7725941

[acel13700-bib-0011] Bárcena, C. , Valdés‐Mas, R. , Mayoral, P. , Garabaya, C. , Durand, S. , Rodríguez, F. , Fernández‐García, M. T. , Salazar, N. , Nogacka, A. M. , Garatachea, N. , Bossut, N. , Aprahamian, F. , Lucia, A. , Kroemer, G. , Freije, J. M. P. , Quirós, P. M. , & López‐Otín, C. (2019). Healthspan and lifespan extension by fecal microbiota transplantation into progeroid mice. Nature Medicine, 25(8), 1234–1242. 10.1038/s41591-019-0504-5 31332389

[acel13700-bib-0012] Barker, N. , van Es, J. H. , Kuipers, J. , Kujala, P. , van den Born, M. , Cozijnsen, M. , Haegebarth, A. , Korving, J. , Begthel, H. , Peters, P. J. , & Clevers, H. (2007). Identification of stem cells in small intestine and colon by marker gene Lgr5. Nature, 449(7165), 1003–1007. 17934449 10.1038/nature06196

[acel13700-bib-0013] Barzilai, N. , Huffman, D. M. , Muzumdar, R. H. , & Bartke, A. (2012). The critical role of metabolic pathways in aging. Diabetes, 61(6), 1315–1322. 22618766 10.2337/db11-1300 PMC3357299

[acel13700-bib-0014] Basile, G. , Paffumi, I. , D'Angelo, A. G. , Figliomeni, P. , Cucinotta, M. D. , Pace, E. , Ferraro, M. , Saitta, S. , Lasco, A. , & Gangemi, S. (2012). Healthy centenarians show high levels of circulating interleukin‐22 (IL‐22). Archives of Gerontology and Geriatrics, 54(3), 459–461. 21640395 10.1016/j.archger.2011.05.004

[acel13700-bib-0015] Belkaid, Y. , & Hand, T. W. (2014). Role of the microbiota in immunity and inflammation. Cell, 157(1), 121–141. 24679531 10.1016/j.cell.2014.03.011 PMC4056765

[acel13700-bib-0016] Bergström, A. , Kristensen, M. B. , Bahl, M. I. , Metzdorff, S. B. , Fink, L. N. , Frøkiaer, H. , & Licht, T. R. (2012). Nature of bacterial colonization influences transcription of mucin genes in mice during the first week of life. BMC Research Notes, 5, 402. 22857743 10.1186/1756-0500-5-402 PMC3465226

[acel13700-bib-0017] Bergstrom, K. S. , Morampudi, V. , Chan, J. M. , Bhinder, G. , Lau, J. , Yang, H. , Ma, C. , Huang, T. , Ryz, N. , Sham, H. P. , Zarepour, M. , Zaph, C. , Artis, D. , Nair, M. , & Vallance, B. A. (2015). Goblet cell derived RELM‐β recruits CD4^+^ T cells during infectious colitis to promote protective intestinal epithelial cell proliferation. PLoS Pathogens, 11(8), e1005108. 26285214 10.1371/journal.ppat.1005108 PMC4540480

[acel13700-bib-0018] Biagi, E. , Franceschi, C. , Rampelli, S. , Severgnini, M. , Ostan, R. , Turroni, S. , Consolandi, C. , Quercia, S. , Scurti, M. , Monti, D. , Capri, M. , Brigidi, P. , & Candela, M. (2016). Gut microbiota and extreme longevity. Current Biology, 26(11), 1480–1485. 27185560 10.1016/j.cub.2016.04.016

[acel13700-bib-0019] Biagi, E. , Nylund, L. , Candela, M. , Ostan, R. , Bucci, L. , Pini, E. , Nikkïla, J. , Monti, D. , Satokari, R. , Franceschi, C. , Brigidi, P. , & de Vos, W. (2010). Through ageing, and beyond: Gut microbiota and inflammatory status in seniors and centenarians. PLoS One, 5(5), e10667. 20498852 10.1371/journal.pone.0010667 PMC2871786

[acel13700-bib-0020] Binyamin, D. , Werbner, N. , Nuriel‐Ohayon, M. , Uzan, A. , Mor, H. , Abbas, A. , Ziv, O. , Teperino, R. , Gutman, R. , & Koren, O. (2020). The aging mouse microbiome has obesogenic characteristics. Genome Medicine, 12(1), 87. 33046129 10.1186/s13073-020-00784-9 PMC7552538

[acel13700-bib-0021] Biton, M. , Haber, A. L. , Rogel, N. , Burgin, G. , Beyaz, S. , Schnell, A. , Ashenberg, O. , Su, C. W. , Smillie, C. , Shekhar, K. , Chen, Z. , Wu, C. , Ordovas‐Montanes, J. , Alvarez, D. , Herbst, R. H. , Zhang, M. , Tirosh, I. , Dionne, D. , Nguyen, L. T. , … Xavier, R. J. (2018). T helper cell cytokines modulate intestinal stem cell renewal and differentiation. Cell, 175(5), 1307–20.e22. 30392957 10.1016/j.cell.2018.10.008 PMC6239889

[acel13700-bib-0022] Bodogai, M. , O'Connell, J. , Kim, K. , Kim, Y. , Moritoh, K. , Chen, C. , Gusev, F. , Vaughan, K. , Shulzhenko, N. , Mattison, J. A. , Lee‐Chang, C. , Chen, W. , Carlson, O. , Becker, K. G. , Gurung, M. , Morgun, A. , White, J. , Meade, T. , Perdue, K. , … Biragyn, A. (2018). Commensal bacteria contribute to insulin resistance in aging by activating innate B1a cells. Science Translational Medicine, 10(467), eaat4271. 30429354 10.1126/scitranslmed.aat4271 PMC6445267

[acel13700-bib-0023] Boehme, M. , Guzzetta, K. E. , Bastiaanssen, T. F. S. , van de Wouw, M. , Moloney, G. M. , Gual‐Grau, A. , Spichak, S. , Olavarría‐Ramírez, L. , Fitzgerald, P. , Morillas, E. , Ritz, N. L. , Jaggar, M. , Cowan, C. S. M. , Crispie, F. , Donoso, F. , Halitzki, E. , Neto, M. C. , Sichetti, M. , Golubeva, A. V. , … Cryan, J. F. (2021). Microbiota from young mice counteracts selective age‐associated behavioral deficits. Nature Aging, 1(8), 666–676. 10.1038/s43587-021-00093-9 37117767

[acel13700-bib-0024] Boehmer, E. D. , Meehan, M. J. , Cutro, B. T. , & Kovacs, E. J. (2005). Aging negatively skews macrophage TLR2‐ and TLR4‐mediated pro‐inflammatory responses without affecting the IL‐2‐stimulated pathway. Mechanisms of Ageing and Development, 126(12), 1305–1313. 16154177 10.1016/j.mad.2005.07.009

[acel13700-bib-0025] Bosco, N. , & Noti, M. (2021). The aging gut microbiome and its impact on host immunity. Genes & Immunity, 22(5), 289–303. 33875817 10.1038/s41435-021-00126-8 PMC8054695

[acel13700-bib-0026] Broderick, N. A. , Buchon, N. , & Lemaitre, B. (2014). Microbiota‐induced changes in *Drosophila melanogaster* host gene expression and gut morphology. MBio, 5(3), e01117‐14. 10.1128/mBio.01117-14 PMC4045073 24865556

[acel13700-bib-0027] Bruunsgaard, H. , Andersen‐Ranberg, K. , Hjelmborg, J. , Pedersen, B. K. , & Jeune, B. (2003). Elevated levels of tumor necrosis factor alpha and mortality in centenarians. The American Journal of Medicine, 115(4), 278–283. 12967692 10.1016/s0002-9343(03)00329-2

[acel13700-bib-0028] Bry, L. , Falk, P. , Huttner, K. , Ouellette, A. , Midtvedt, T. , & Gordon, J. I. (1994). Paneth cell differentiation in the developing intestine of normal and transgenic mice. Proceedings of the National Academy of Sciences of the United States of America, 91(22), 10335–10339. 7937951 10.1073/pnas.91.22.10335 PMC45014

[acel13700-bib-0029] Bunker, J. J. , & Bendelac, A. (2018). IgA responses to microbiota. Immunity, 49(2), 211–224. 30134201 10.1016/j.immuni.2018.08.011 PMC6107312

[acel13700-bib-0030] Catanzaro, J. R. , Strauss, J. D. , Bielecka, A. , Porto, A. F. , Lobo, F. M. , Urban, A. , Schofield, W. B. , & Palm, N. W. (2019). IgA‐deficient humans exhibit gut microbiota dysbiosis despite secretion of compensatory IgM. Scientific Reports, 9(1), 13574. 31537840 10.1038/s41598-019-49923-2 PMC6753154

[acel13700-bib-0031] Chandrakesan, P. , May, R. , Weygant, N. , Qu, D. , Berry, W. L. , Sureban, S. M. , Ali, N. , Rao, C. , Huycke, M. , Bronze, M. S. , & Houchen, C. W. (2016). Intestinal tuft cells regulate the ATM mediated DNA Damage response via Dclk1 dependent mechanism for crypt restitution following radiation injury. Scientific Reports, 6(1), 37667. 27876863 10.1038/srep37667 PMC5120335

[acel13700-bib-0032] Channappanavar, R. , Twardy, B. S. , Krishna, P. , & Suvas, S. (2009). Advancing age leads to predominance of inhibitory receptor expressing CD4 T cells. Mechanisms of Ageing and Development, 130(10), 709–712. 19715717 10.1016/j.mad.2009.08.006

[acel13700-bib-0033] Cheng, H. , & Leblond, C. P. (1974). Origin, differentiation and renewal of the four main epithelial cell types in the mouse small intestine. V. Unitarian Theory of the origin of the four epithelial cell types. The American Journal of Anatomy, 141(4), 537–561. 4440635 10.1002/aja.1001410407

[acel13700-bib-0034] Cho, R. H. , Sieburg, H. B. , & Muller‐Sieburg, C. E. (2008). A new mechanism for the aging of hematopoietic stem cells: aging changes the clonal composition of the stem cell compartment but not individual stem cells. Blood, 111(12), 5553–5561. 18413859 10.1182/blood-2007-11-123547 PMC2424153

[acel13700-bib-0035] Chung, H. , Pamp, S. J. , Hill, J. A. , Surana, N. K. , Edelman, S. M. , Troy, E. B. , Reading, N. C. , Villablanca, E. J. , Wang, S. , Mora, J. R. , Umesaki, Y. , Mathis, D. , Benoist, C. , Relman, D. A. , & Kasper, D. L. (2012). Gut immune maturation depends on colonization with a host‐specific microbiota. Cell, 149(7), 1578–1593. 22726443 10.1016/j.cell.2012.04.037 PMC3442780

[acel13700-bib-0036] Ciabattini, A. , Nardini, C. , Santoro, F. , Garagnani, P. , Franceschi, C. , & Medaglini, D. (2018). Vaccination in the elderly: The challenge of immune changes with aging. Seminars in Immunology, 40, 83–94. 30501873 10.1016/j.smim.2018.10.010

[acel13700-bib-0037] Ciccocioppo, R. , Di Sabatino, A. , Luinetti, O. , Rossi, M. , Cifone, M. G. , & Corazza, G. R. (2002). Small bowel enterocyte apoptosis and proliferation are increased in the elderly. Gerontology, 48(4), 204–208. 12053108 10.1159/000058351

[acel13700-bib-0038] Claesson, M. J. , Jeffery, I. B. , Conde, S. , Power, S. E. , O'Connor, E. M. , Cusack, S. , Harris, H. M. , Coakley, M. , Lakshminarayanan, B. , O'Sullivan, O. , Fitzgerald, G. F. , Deane, J. , O'Connor, M. , Harnedy, N. , O'Connor, K. , O'Mahony, D. , van Sinderen, D. , Wallace, M. , Brennan, L. , … O'Toole, P. W. (2012). Gut microbiota composition correlates with diet and health in the elderly. Nature, 488(7410), 178–184. 22797518 10.1038/nature11319

[acel13700-bib-0039] Clark, R. I. , Salazar, A. , Yamada, R. , Fitz‐Gibbon, S. , Morselli, M. , Alcaraz, J. , Rana, A. , Rera, M. , Pellegrini, M. , Ja, W. W. , & Walker, D. W. (2015). Distinct shifts in microbiota composition during *Drosophila* aging impair intestinal function and drive mortality. Cell Reports, 12(10), 1656–1667. 26321641 10.1016/j.celrep.2015.08.004 PMC4565751

[acel13700-bib-0040] Clevers, H. (2013). The intestinal crypt, a prototype stem cell compartment. Cell, 154(2), 274–284. 23870119 10.1016/j.cell.2013.07.004

[acel13700-bib-0041] Collino, S. , Montoliu, I. , Martin, F. P. , Scherer, M. , Mari, D. , Salvioli, S. , Bucci, L. , Ostan, R. , Monti, D. , Biagi, E. , Brigidi, P. , Franceschi, C. , & Rezzi, S. (2013). Metabolic signatures of extreme longevity in northern Italian centenarians reveal a complex remodeling of lipids, amino acids, and gut microbiota metabolism. PLoS One, 8(3), e56564. 23483888 10.1371/journal.pone.0056564 PMC3590212

[acel13700-bib-0042] Conley, M. N. , Wong, C. P. , Duyck, K. M. , Hord, N. , Ho, E. , & Sharpton, T. J. (2016). Aging and serum MCP‐1 are associated with gut microbiome composition in a murine model. PeerJ, 4, e1854. 27069796 10.7717/peerj.1854 PMC4824877

[acel13700-bib-0043] Corrêa‐Oliveira, R. , Fachi, J. L. , Vieira, A. , Sato, F. T. , & Vinolo, M. A. R. (2016). Regulation of immune cell function by short‐chain fatty acids. Clinical & Translational Immunology, 5(4), e73. 27195116 10.1038/cti.2016.17 PMC4855267

[acel13700-bib-0044] Cui, H. , Tang, D. , Garside, G. B. , Zeng, T. , Wang, Y. , Tao, Z. , Zhang, L. , & Tao, S. (2019). Wnt signaling mediates the aging‐induced differentiation impairment of intestinal stem cells. Stem Cell Reviews and Reports, 15(3), 448–455. 30790135 10.1007/s12015-019-09880-9 PMC6534527

[acel13700-bib-0045] D'Amato, A. , di Cesare Mannelli, L. , Lucarini, E. , Man, A. L. , le Gall, G. , Branca, J. J. V. , Ghelardini, C. , Amedei, A. , Bertelli, E. , Regoli, M. , Pacini, A. , Luciani, G. , Gallina, P. , Altera, A. , Narbad, A. , Gulisano, M. , Hoyles, L. , Vauzour, D. , & Nicoletti, C. (2020). Faecal microbiota transplant from aged donor mice affects spatial learning and memory via modulating hippocampal synaptic plasticity‐ and neurotransmission‐related proteins in young recipients. Microbiome, 8(1), 140. 33004079 10.1186/s40168-020-00914-w PMC7532115

[acel13700-bib-0046] David, L. A. , Maurice, C. F. , Carmody, R. N. , Gootenberg, D. B. , Button, J. E. , Wolfe, B. E. , Ling, A. V. , Devlin, A. S. , Varma, Y. , Fischbach, M. A. , Biddinger, S. B. , Dutton, R. J. , & Turnbaugh, P. J. (2014). Diet rapidly and reproducibly alters the human gut microbiome. Nature, 505(7484), 559–563. 24336217 10.1038/nature12820 PMC3957428

[acel13700-bib-0047] de Jesús Garduno‐Garcia, J. , Gastaldelli, A. , DeFronzo, R. A. , Lertwattanarak, R. , Holst, J. J. , & Musi, N. (2018). Older subjects with β‐cell dysfunction have an accentuated incretin release. The Journal of Clinical Endocrinology & Metabolism, 103(7), 2613–2619. 29672742 10.1210/jc.2018-00260 PMC6669818

[acel13700-bib-0048] de Maeyer, R. P. H. , van de Merwe, R. C. , Louie, R. , Bracken, O. V. , Devine, O. P. , Goldstein, D. R. , Uddin, M. , Akbar, A. N. , & Gilroy, D. W. (2020). Blocking elevated p38 MAPK restores efferocytosis andinflammatory resolution in the elderly. Nature Immunology, 21(6), 615–625. 32251403 10.1038/s41590-020-0646-0 PMC7983074

[acel13700-bib-0049] Decman, V. , Laidlaw, B. J. , Doering, T. A. , Leng, J. , Ertl, H. C. , Goldstein, D. R. , & Wherry, E. J. (2012). Defective CD8 T cell responses in aged mice are due to quantitative and qualitative changes in virus‐specific precursors. Journal of Immunology, 188(4), 1933–1941. 10.4049/jimmunol.1101098 PMC3320034 22246631

[acel13700-bib-0050] Deleidi, M. , Jäggle, M. , & Rubino, G. (2015). Immune aging, dysmetabolism, and inflammation in neurological diseases. Frontiers in Neuroscience, 9, 172. 26089771 10.3389/fnins.2015.00172 PMC4453474

[acel13700-bib-0051] Della Bella, S. , Bierti, L. , Presicce, P. , Arienti, R. , Valenti, M. , Saresella, M. , Vergani, C. , & Villa, M. L. (2007). Peripheral blood dendritic cells and monocytes are differently regulated in the elderly. Clinical Immunology (Orlando, Fla)., 122(2), 220–228. 10.1016/j.clim.2006.09.012 17101294

[acel13700-bib-0052] Dignass, A. , Lynch‐Devaney, K. , Kindon, H. , Thim, L. , & Podolsky, D. K. (1994). Trefoil peptides promote epithelial migration through a transforming growth factor beta‐independent pathway. The Journal of Clinical Investigation, 94(1), 376–383. 8040278 10.1172/JCI117332 PMC296319

[acel13700-bib-0053] Donaldson, D. S. , Pollock, J. , Vohra, P. , Stevens, M. P. , & Mabbott, N. A. (2020). Microbial stimulation reverses the age‐related decline in M cells in aged mice. iScience, 23(6), 101147. 32454449 10.1016/j.isci.2020.101147 PMC7251786

[acel13700-bib-0054] Drago, L. , Toscano, M. , Rodighiero, V. , De Vecchi, E. , & Mogna, G. (2012). Cultivable and pyrosequenced fecal microflora in centenarians and young subjects. Journal of Clinical Gastroenterology, 46, S81–S84. 22955365 10.1097/MCG.0b013e3182693982

[acel13700-bib-0055] Duan, L. , Chen, J. , Zhang, H. , Yang, H. , Zhu, P. , Xiong, A. , Xia, Q. , Zheng, F. , Tan, Z. , Gong, F. , & Fang, M. (2012). Interleukin‐33 ameliorates experimental colitis through promoting Th2/Foxp3^+^ regulatory T‐cell responses in mice. Molecular Medicine, 18(5), 753–761. 22426954 10.2119/molmed.2011.00428 PMC3409280

[acel13700-bib-0056] Dunn‐Walters, D. K. (2016). The ageing human B cell repertoire: A failure of selection? Clinical & Experimental Immunology, 183(1), 50–56. 26332693 10.1111/cei.12700 PMC4687518

[acel13700-bib-0057] Egerod, K. L. , Engelstoft, M. S. , Grunddal, K. V. , Nøhr, M. K. , Secher, A. , Sakata, I. , Pedersen, J. , Windeløv, J. A. , Füchtbauer, E. M. , Olsen, J. , Sundler, F. , Christensen, J. P. , Wierup, N. , Olsen, J. V. , Holst, J. J. , Zigman, J. M. , Poulsen, S. S. , & Schwartz, T. W. (2012). A major lineage of enteroendocrine cells coexpress CCK, secretin, GIP, GLP‐1, PYY, and neurotensin but not somatostatin. Endocrinology, 153(12), 5782–5795. 23064014 10.1210/en.2012-1595 PMC7958714

[acel13700-bib-0058] Elderman, M. , Sovran, B. , Hugenholtz, F. , Graversen, K. , Huijskes, M. , Houtsma, E. , Belzer, C. , Boekschoten, M. , de Vos, P. , Dekker, J. , Wells, J. , & Faas, M. (2017). The effect of age on the intestinal mucus thickness, microbiota composition and immunity in relation to sex in mice. PLoS One, 12(9), e0184274. 28898292 10.1371/journal.pone.0184274 PMC5595324

[acel13700-bib-0059] Erny, D. , Hrabě de Angelis, A. L. , Jaitin, D. , Wieghofer, P. , Staszewski, O. , David, E. , Keren‐Shaul, H. , Mahlakoiv, T. , Jakobshagen, K. , Buch, T. , Schwierzeck, V. , Utermöhlen, O. , Chun, E. , Garrett, W. S. , McCoy, K. , Diefenbach, A. , Staeheli, P. , Stecher, B. , Amit, I. , & Prinz, M. (2015). Host microbiota constantly control maturation and function of microglia in the CNS. Nature Neuroscience, 18(7), 965–977. 26030851 10.1038/nn.4030 PMC5528863

[acel13700-bib-0060] Filyk, H. A. , & Osborne, L. C. (2016). The multibiome: The intestinal ecosystem's influence on immune homeostasis, health, and disease. eBioMedicine, 13, 46–54. 27863931 10.1016/j.ebiom.2016.10.007 PMC5264270

[acel13700-bib-0061] Fox, S. H. , Chuang, R. , & Brotchie, J. M. (2009). Serotonin and Parkinson's disease: On movement, mood, and madness. Movement Disorders: Official Journal of the Movement Disorder Society, 24(9), 1255–1266. 19412960 10.1002/mds.22473

[acel13700-bib-0062] Franceschi, C. , Bonafè, M. , Valensin, S. , Olivieri, F. , De Luca, M. , Ottaviani, E. , & De Benedictis, G. (2000). Inflamm‐aging. An evolutionary perspective on immunosenescence. Annals of the New York Academy of Sciences, 908, 244–254. 10911963 10.1111/j.1749-6632.2000.tb06651.x

[acel13700-bib-0063] Franceschi, C. , Garagnani, P. , Parini, P. , Giuliani, C. , & Santoro, A. (2018). Inflammaging: A new immune–metabolic viewpoint for age‐related diseases. Nature Reviews Endocrinology, 14(10), 576–590. 10.1038/s41574-018-0059-4 30046148

[acel13700-bib-0064] Fransen, F. , van Beek, A. A. , Borghuis, T. , Aidy, S. E. , Hugenholtz, F. , van der Gaast‐de Jongh, C. , HFJ, S. , & De Jonge, M. I. (2017). Aged gut microbiota contributes to systemical inflammaging after transfer to germ‐free mice. Frontiers in Immunology, 8, 1385. 29163474 10.3389/fimmu.2017.01385 PMC5674680

[acel13700-bib-0065] Frasca, D. , & Blomberg, B. B. (2009). Effects of aging on B cell function. Current Opinion in Immunology., 21(4), 425–430. 19608393 10.1016/j.coi.2009.06.001 PMC2853364

[acel13700-bib-0066] Furuse, M. , Fujita, K. , Hiiragi, T. , Fujimoto, K. , & Tsukita, S. (1998). Claudin‐1 and ‐2: Novel integral membrane proteins localizing at tight junctions with no sequence similarity to occludin. Journal of Cell Biology, 141(7), 1539–1550. 9647647 10.1083/jcb.141.7.1539 PMC2132999

[acel13700-bib-0067] Furuse, M. , Hirase, T. , Itoh, M. , Nagafuchi, A. , Yonemura, S. , Tsukita, S. , & Tsukita, S. (1993). Occludin: A novel integral membrane protein localizing at tight junctions. Journal of Cell Biology, 123(6), 1777–1788. 8276896 10.1083/jcb.123.6.1777 PMC2290891

[acel13700-bib-0068] Furuse, M. , Sasaki, H. , Fujimoto, K. , & Tsukita, S. (1998). A single gene product, claudin‐1 or −2, reconstitutes tight junction strands and recruits occludin in fibroblasts. Journal of Cell Biology, 143(2), 391–401. 9786950 10.1083/jcb.143.2.391 PMC2132845

[acel13700-bib-0069] Gangemi, S. , Basile, G. , Merendino, R. A. , Minciullo, P. L. , Novick, D. , Rubinstein, M. , Dinarello, C. A. , Lo Balbo, C. , Franceschi, C. , Basili, S. , D' Urbano, E. , Daví, G. , Nicita‐Mauro, V. , & Romano, M. (2003). Increased circulating Interleukin‐18 levels in centenarians with no signs of vascular disease: Another paradox of longevity? Experimental Gerontology, 38(6), 669–672. 12814802 10.1016/s0531-5565(03)00061-5

[acel13700-bib-0070] Gangemi, S. , Basile, G. , Monti, D. , Merendino, R. A. , Di Pasquale, G. , Bisignano, U. , Nicita‐Mauro, V. , & Franceschi, C. (2005). Age‐related modifications in circulating IL‐15 levels in humans. Mediators of Inflammation, 2005(4), 245–247. 16192677 10.1155/MI.2005.245 PMC1526477

[acel13700-bib-0071] Gerbe, F. , Brulin, B. , Makrini, L. , Legraverend, C. , & Jay, P. (2009). DCAMKL‐1 expression identifies Tuft cells rather than stem cells in the adult mouse intestinal epithelium. Gastroenterology, 137(6), 2179–2180 author reply 80‐1. 19879217 10.1053/j.gastro.2009.06.072

[acel13700-bib-0072] Gerbe, F. , Sidot, E. , Smyth, D. J. , Ohmoto, M. , Matsumoto, I. , Dardalhon, V. , Cesses, P. , Garnier, L. , Pouzolles, M. , Brulin, B. , Bruschi, M. , Harcus, Y. , Zimmermann, V. S. , Taylor, N. , Maizels, R. M. , & Jay, P. (2016). Intestinal epithelial tuft cells initiate type 2 mucosal immunity to helminth parasites. Nature, 529(7585), 226–230. 26762460 10.1038/nature16527 PMC7614903

[acel13700-bib-0073] Gibson, K. L. , Wu, Y. C. , Barnett, Y. , Duggan, O. , Vaughan, R. , Kondeatis, E. , Nilsson, B. O. , Wikby, A. , Kipling, D. , & Dunn‐Walters, D. K. (2009). B‐cell diversity decreases in old age and is correlated with poor health status. Aging Cell, 8(1), 18–25. 18986373 10.1111/j.1474-9726.2008.00443.x PMC2667647

[acel13700-bib-0074] Giovannini, S. , onder, G. , Liperoti, R. , Russo, A. , Carter, C. , Capoluongo, E. , Pahor, M. , Bernabei, R. , & Landi, F. (2011). Interleukin‐6, C‐reactive protein, and tumor necrosis factor‐alpha as predictors of mortality in frail, community‐living elderly individuals. Journal of the American Geriatrics Society, 59(9), 1679–1685. 21883115 10.1111/j.1532-5415.2011.03570.x PMC4321727

[acel13700-bib-0075] Golomb, S. M. , Guldner, I. H. , Zhao, A. , Wang, Q. , Palakurthi, B. , Aleksandrovic, E. A. , Lopez, J. A. , Lee, S. W. , Yang, K. , & Zhang, S. (2020). Multi‐modal single‐cell analysis reveals brain immune landscape plasticity during aging and gut microbiota dysbiosis. Cell Reports, 33(9), 108438. 33264626 10.1016/j.celrep.2020.108438 PMC7737488

[acel13700-bib-0076] Goodrich, J. K. , Waters, J. L. , Poole, A. C. , Sutter, J. L. , Koren, O. , Blekhman, R. , Beaumont, M. , Van Treuren, W. , Knight, R. , Bell, J. T. , Spector, T. D. , Clark, A. G. , & Ley, R. E. (2014). Human genetics shape the gut microbiome. Cell, 159(4), 789–799. 25417156 10.1016/j.cell.2014.09.053 PMC4255478

[acel13700-bib-0077] Gopinath, B. , Kifley, A. , Flood, V. M. , & Mitchell, P. (2018). Physical activity as a determinant of successful aging over ten years. Scientific Reports, 8(1), 10522. 30002462 10.1038/s41598-018-28526-3 PMC6043510

[acel13700-bib-0078] Goronzy, J. J. , & Weyand, C. M. (2012). Immune aging and autoimmunity. Cellular and Molecular Life Sciences: CMLS, 69(10), 1615–1623. 22466672 10.1007/s00018-012-0970-0 PMC4277694

[acel13700-bib-0079] Grajeda‐Iglesias, C. , Durand, S. , Daillère, R. , Iribarren, K. , Lemaitre, F. , Derosa, L. , Aprahamian, F. , Bossut, N. , Nirmalathasan, N. , Madeo, F. , Zitvogel, L. , & Kroemer, G. (2021). Oral administration of *Akkermansia muciniphila* elevates systemic antiaging and anticancer metabolites. Aging, 13(5), 6375–6405. 33653967 10.18632/aging.202739 PMC7993698

[acel13700-bib-0080] Grunddal, K. V. , Ratner, C. F. , Svendsen, B. , Sommer, F. , Engelstoft, M. S. , Madsen, A. N. , Pedersen, J. , Nøhr, M. K. , Egerod, K. L. , Nawrocki, A. R. , Kowalski, T. , Howard, A. D. , Poulsen, S. S. , Offermanns, S. , Bäckhed, F. , Holst, J. J. , Holst, B. , & Schwartz, T. W. (2016). Neurotensin is coexpressed, coreleased, and acts together with GLP‐1 and PYY in enteroendocrine control of metabolism. Endocrinology, 157(1), 176–194. 26469136 10.1210/en.2015-1600

[acel13700-bib-0081] Gustafson, C. E. , Kim, C. , Weyand, C. M. , & Goronzy, J. J. (2020). Influence of immune aging on vaccine responses. The Journal of Allergy and Clinical Immunology, 145(5), 1309–1321. 32386655 10.1016/j.jaci.2020.03.017 PMC7198995

[acel13700-bib-0082] Habib, S. , El Andaloussi, A. , Elmasry, K. , Handoussa, A. , Azab, M. , Elsawey, A. , Al‐Hendy, A. , & Ismail, N. (2018). PDL‐1 blockade prevents T cell exhaustion, inhibits autophagy, and promotes clearance of *Leishmania donovani* . Infection and Immunity, 86(6), e00019‐18. 29610255 10.1128/IAI.00019-18 PMC5964517

[acel13700-bib-0083] Hagan, T. , Cortese, M. , Rouphael, N. , Boudreau, C. , Linde, C. , Maddur, M. S. , das, J. , Wang, H. , Guthmiller, J. , Zheng, N. Y. , Huang, M. , Uphadhyay, A. A. , Gardinassi, L. , Petitdemange, C. , McCullough, M. , Johnson, S. J. , Gill, K. , Cervasi, B. , Zou, J. , … Pulendran, B. (2019). Antibiotics‐driven gut microbiome perturbation alters immunity to vaccines in humans. Cell, 178(6), 1313–28.e13. 31491384 10.1016/j.cell.2019.08.010 PMC6750738

[acel13700-bib-0084] Hall, B. M. , Balan, V. , Gleiberman, A. S. , Strom, E. , Krasnov, P. , Virtuoso, L. P. , Rydkina, E. , Vujcic, S. , Balan, K. , Gitlin, I. , Leonova, K. , Polinsky, A. , Chernova, O. B. , & Gudkov, A. V. (2016). Aging of mice is associated with p16(Ink4a)‐ and β‐galactosidase‐positive macrophage accumulation that can be induced in young mice by senescent cells. Aging, 8(7), 1294–1315. 27391570 10.18632/aging.100991 PMC4993332

[acel13700-bib-0085] He, W. , Wang, M. L. , Jiang, H. Q. , Steppan, C. M. , Shin, M. E. , Thurnheer, M. C. , Cebra, J. J. , Lazar, M. A. , & Wu, G. D. (2003). Bacterial colonization leads to the colonic secretion of RELMbeta/FIZZ2, a novel goblet cell‐specific protein. Gastroenterology, 125(5), 1388–1397. 14598255 10.1016/j.gastro.2003.07.009

[acel13700-bib-0086] Hearps, A. C. , Martin, G. E. , Angelovich, T. A. , Cheng, W.‐J. , Maisa, A. , Landay, A. L. , Jaworowski, A. , & Crowe, S. M. (2012). Aging is associated with chronic innate immune activation and dysregulation of monocyte phenotype and function. Aging Cell, 11(5), 867–875. 22708967 10.1111/j.1474-9726.2012.00851.x

[acel13700-bib-0087] Heazlewood, C. K. , Cook, M. C. , Eri, R. , Price, G. R. , Tauro, S. B. , Taupin, D. , Thornton, D. J. , Png, C. W. , Crockford, T. L. , Cornall, R. J. , Adams, R. , Kato, M. , Nelms, K. A. , Hong, N. A. , Florin, T. H. , Goodnow, C. C. , & McGuckin, M. (2008). Aberrant mucin assembly in mice causes endoplasmic reticulum stress and spontaneous inflammation resembling ulcerative colitis. PLoS Medicine, 5(3), e54. 18318598 10.1371/journal.pmed.0050054 PMC2270292

[acel13700-bib-0088] Henrich, T. J. , Krakower, D. , Bitton, A. , & Yokoe, D. S. (2009). Clinical risk factors for severe *Clostridium difficile*‐associated disease. Emerging Infectious Diseases, 15(3), 415–422. 19239754 10.3201/eid1503.080312 PMC2681109

[acel13700-bib-0089] Hooper, L. V. , & Macpherson, A. J. (2010). Immune adaptations that maintain homeostasis with the intestinal microbiota. Nature Reviews Immunology, 10(3), 159–169. 10.1038/nri2710 20182457

[acel13700-bib-0090] Howitt, M. R. , Lavoie, S. , Michaud, M. , Blum, A. M. , Tran, S. V. , Weinstock, J. V. , Gallini, C. A. , Redding, K. , Margolskee, R. F. , Osborne, L. C. , Artis, D. , & Garrett, W. S. (2016). Tuft cells, taste‐chemosensory cells, orchestrate parasite type 2 immunity in the gut. Science, 351(6279), 1329–1333. 26847546 10.1126/science.aaf1648 PMC5528851

[acel13700-bib-0091] Ichinohe, T. , Pang, I. K. , Kumamoto, Y. , Peaper, D. R. , Ho, J. H. , Murray, T. S. , & Iwasaki, A. (2011). Microbiota regulates immune defense against respiratory tract influenza A virus infection. Proceedings of the National Academy of Sciences of the United States of America, 108(13), 5354–5359. 21402903 10.1073/pnas.1019378108 PMC3069176

[acel13700-bib-0092] Ikeguchi, E. , Harada, N. , Kanemaru, Y. , Sankoda, A. , Yamane, S. , Iwasaki, K. , Imajo, M. , Murata, Y. , Suzuki, K. , Joo, E. , & Inagaki, N. (2018). Transcriptional factor Pdx1 is involved in age‐related GIP hypersecretion in mice. American Journal of Physiology Gastrointestinal and Liver Physiology, 315(2), G272–G282. 29723041 10.1152/ajpgi.00054.2018

[acel13700-bib-0093] Jackson, M. A. , Jeffery, I. B. , Beaumont, M. , Bell, J. T. , Clark, A. G. , Ley, R. E. , O'Toole, P. W. , Spector, T. D. , & Steves, C. J. (2016). Signatures of early frailty in the gut microbiota. Genome Medicine, 8(1), 8. 26822992 10.1186/s13073-016-0262-7 PMC4731918

[acel13700-bib-0094] Julliard, W. , De Wolfe, T. J. , Fechner, J. H. , Safdar, N. , Agni, R. , & Mezrich, J. D. (2017). Amelioration of *Clostridium difficile* infection in mice by dietary supplementation with indole‐3‐carbinol. Annals of Surgery, 265(6), 1183–1191. 27280500 10.1097/SLA.0000000000001830 PMC5743052

[acel13700-bib-0095] Kanemaru, Y. , Harada, N. , Shimazu‐Kuwahara, S. , Yamane, S. , Ikeguchi, E. , Murata, Y. , Kiyobayashi, S. , Hatoko, T. , & Inagaki, N. (2020). Absence of GIP secretion alleviates age‐related obesity and insulin resistance. The Journal of Endocrinology, 245(1), 13–20. 31977316 10.1530/JOE-19-0477 PMC7040458

[acel13700-bib-0096] Kato, H. , Fujihashi, K. , Kato, R. , Dohi, T. , Fujihashi, K. , Hagiwara, Y. , Kataoka, K. , Kobayashi, R. , & McGhee, J. (2003). Lack of oral tolerance in aging is due to sequential loss of Peyer's patch cell interactions. International Immunology, 15(2), 145–158. 12578844 10.1093/intimm/dxg011

[acel13700-bib-0097] Khan, S. R. , van der Burgh, A. C. , Peeters, R. P. , van Hagen, P. M. , Dalm, V. A. S. H. , & Chaker, L. (2021). Determinants of serum immunoglobulin levels. A systematic review and meta‐analysis. Frontiers in Immunology, 12(1103), 664526. 33897714 10.3389/fimmu.2021.664526 PMC8058410

[acel13700-bib-0098] Kobayashi, A. , Donaldson, D. S. , Erridge, C. , Kanaya, T. , Williams, I. R. , Ohno, H. , Mahajan, A. , & Mabbott, N. A. (2013). The functional maturation of M cells is dramatically reduced in the Peyer's patches of aged mice. Mucosal Immunology, 6(5), 1027–1037. 23360902 10.1038/mi.2012.141 PMC3747980

[acel13700-bib-0099] Koga, T. , McGhee, J. R. , Kato, H. , Kato, R. , Kiyono, H. , & Fujihashi, K. (2000). Evidence for early aging in the mucosal immune system. Journal of Immunology, 165(9), 5352–5359. 10.4049/jimmunol.165.9.5352 11046071

[acel13700-bib-0100] Kovtonyuk, L. V. , Caiado, F. , Garcia‐Martin, S. , Manz, E. M. , Helbling, P. , Takizawa, H. , Boettcher, S. , Al‐Shahrour, F. , Nombela‐Arrieta, C. , Slack, E. , & Manz, M. G. (2021). IL‐1 mediates microbiome‐induced inflamm‐ageing of hematopoietic stem cells in mice. Blood, 139, 44–58. 10.1182/blood.2021011570 34525198

[acel13700-bib-0101] Krimi, R. B. , Kotelevets, L. , Dubuquoy, L. , Plaisancié, P. , Walker, F. , Lehy, T. , Desreumaux, P. , van Seuningen, I. , Chastre, E. , Forgue‐Lafitte, M. E. , & Marie, J. C. (2008). Resistin‐like molecule β regulates intestinal mucous secretion and curtails TNBS‐induced colitis in mice. Inflammatory Bowel Diseases, 14(7), 931–941. 18300276 10.1002/ibd.20420

[acel13700-bib-0102] Langille, M. G. I. , Meehan, C. J. , Koenig, J. E. , Dhanani, A. S. , Rose, R. A. , Howlett, S. E. , & Beiko, R. G. (2014). Microbial shifts in the aging mouse gut. Microbiome., 2(1), 50. 25520805 10.1186/s40168-014-0050-9 PMC4269096

[acel13700-bib-0103] Lee, J. , Venna, V. R. , Durgan, D. J. , Shi, H. , Hudobenko, J. , Putluri, N. , Petrosino, J. , McCullough, L. , & Bryan, R. M. (2020). Young versus aged microbiota transplants to germ‐free mice: Increased short‐chain fatty acids and improved cognitive performance. Gut Microbes, 12(1), 1–14. 10.1080/19490976.2020.1814107 PMC7757789 32897773

[acel13700-bib-0104] Lee, K. A. , Shin, K. S. , Kim, G. Y. , Song, Y. C. , Bae, E. A. , Kim, I. K. , Koh, C. H. , & Kang, C. Y. (2016). Characterization of age‐associated exhausted CD8^+^ T cells defined by increased expression of Tim‐3 and PD‐1. Aging Cell, 15(2), 291–300. 26750587 10.1111/acel.12435 PMC4783346

[acel13700-bib-0105] Li, Q. , Han, D. , Cong, B. , Shan, B. , Zhang, J. , Chen, H. , Ma, C. , & Liyanage, S. S. (2011). Cholecystokinin octapeptide significantly suppresses collagen‐induced arthritis in mice by inhibiting Th17 polarization primed by dendritic cells. Cellular Immunology, 272(1), 53–60. 22004797 10.1016/j.cellimm.2011.09.007

[acel13700-bib-0106] Li, Y. , Ning, L. , Yin, Y. , Wang, R. , Zhang, Z. , Hao, L. , Wang, B. , Zhao, X. , Yang, X. , Yin, L. , Wu, S. , Guo, D. , & Zhang, C. (2020). Age‐related shifts in gut microbiota contribute to cognitive decline in aged rats. Aging, 12(9), 7801–7817. 32357144 10.18632/aging.103093 PMC7244050

[acel13700-bib-0107] Liang, S. , Domon, H. , Hosur, K. B. , Wang, M. , & Hajishengallis, G. (2009). Age‐related alterations in innate immune receptor expression and ability of macrophages to respond to pathogen challenge *in vitro* . Mechanisms of Ageing and Development, 130(8), 538–546. 19559723 10.1016/j.mad.2009.06.006 PMC2717634

[acel13700-bib-0108] Liguori, I. , Russo, G. , Curcio, F. , Bulli, G. , Aran, L. , Della‐Morte, D. , Gargiulo, G. , Testa, G. , Cacciatore, F. , Bonaduce, D. , & Abete, P. (2018). Oxidative stress, aging, and diseases. Clinical Interventions in Aging, 13, 757–772. 29731617 10.2147/CIA.S158513 PMC5927356

[acel13700-bib-0109] Luan, Z. , Sun, G. , Huang, Y. , Yang, Y. , Yang, R. , Li, C. , Wang, T. , Tan, D. , Qi, S. , Jun, C. , Wang, C. , Wang, S. , Zhao, Y. , & Jing, Y. (2020). Metagenomics study reveals changes in gut microbiota in centenarians: A cohort study of Hainan Centenarians. Frontiers in Microbiology, 11, 1474. 32714309 10.3389/fmicb.2020.01474 PMC7343713

[acel13700-bib-0110] Luo, X.‐C. , Chen, Z.‐H. , Xue, J.‐B. , Zhao, D.‐X. , Lu, C. , Li, Y.‐H. , Li, S. M. , du, Y. W. , Liu, Q. , Wang, P. , Liu, M. , & Huang, L. (2019). Infection by the parasitic helminth *Trichinella spiralis* activates a Tas2r‐mediated signaling pathway in intestinal tuft cells. Proceedings of the National Academy of Sciences of the United States of America, 116(12), 5564–5569. 30819885 10.1073/pnas.1812901116 PMC6431192

[acel13700-bib-0111] Lynn, D. J. , Benson, S. C. , Lynn, M. A. , & Pulendran, B. (2021). Modulation of immune responses to vaccination by the microbiota: implications and potential mechanisms. Nature Reviews Immunology, 22(1), 33–46. 10.1038/s41577-021-00554-7 PMC8127454 34002068

[acel13700-bib-0112] Lynn, D. J. , & Pulendran, B. (2018). The potential of the microbiota to influence vaccine responses. Journal of Leukocyte Biology, 103(2), 225–231. 28864446 10.1189/jlb.5MR0617-216R PMC5921907

[acel13700-bib-0113] Ma, S. , Wang, C. , Mao, X. , & Hao, Y. (2019). B cell dysfunction associated with aging and autoimmune diseases. Frontiers in Immunology, 10, 318. 30873171 10.3389/fimmu.2019.00318 PMC6400972

[acel13700-bib-0114] Mabbott, N. A. , Donaldson, D. S. , Ohno, H. , Williams, I. R. , & Mahajan, A. (2013). Microfold (M) cells: Important immunosurveillance posts in the intestinal epithelium. Mucosal Immunology, 6(4), 666–677. 23695511 10.1038/mi.2013.30 PMC3686595

[acel13700-bib-0115] Macpherson, A. J. , & Uhr, T. (2004). Induction of protective IgA by intestinal dendritic cells carrying commensal bacteria. Science, 303(5664), 1662–1665. 15016999 10.1126/science.1091334

[acel13700-bib-0116] Macpherson, A. J. , Yilmaz, B. , Limenitakis, J. P. , & Ganal‐Vonarburg, S. C. (2018). IgA function in relation to the intestinal microbiota. Annual Review of Immunology, 36(1), 359–381. 10.1146/annurev-immunol-042617-053238 29400985

[acel13700-bib-0117] Magrone, T. , & Jirillo, E. (2013). The interaction between gut microbiota and age‐related changes in immune function and inflammation. Immunity & Ageing: I & A, 10(1), 31. 23915308 10.1186/1742-4933-10-31 PMC3848811

[acel13700-bib-0118] Malinova, T. S. , Dijkstra, C. D. , & de Vries, H. E. (2018). Serotonin: A mediator of the gut‐brain axis in multiple sclerosis. Multiple sclerosis (Houndmills, Basingstoke, England), 24(9), 1144–1150. 10.1177/1352458517739975 PMC6052430 29117778

[acel13700-bib-0119] Man, A. L. , Bertelli, E. , Rentini, S. , Regoli, M. , Briars, G. , Marini, M. , Watson, A. J. , & Nicoletti, C. (2015). Age‐associated modifications of intestinal permeability and innate immunity in human small intestine. Clinical Science (London, England : 1979), 129(7), 515–527. 10.1042/CS20150046 25948052

[acel13700-bib-0120] Marcos‐Pérez, D. , Sánchez‐Flores, M. , Maseda, A. , Lorenzo‐López, L. , Millán‐Calenti, J. C. , Gostner, J. M. , Fuchs, D. , Pásaro, E. , Laffon, B. , & Valdiglesias, V. (2018). Frailty in older adults is associated with plasma concentrations of inflammatory mediators but not with lymphocyte subpopulations. Frontiers in Immunology, 9, 1056. 29868017 10.3389/fimmu.2018.01056 PMC5964167

[acel13700-bib-0121] Martin, C. , Burdon, P. C. E. , Bridger, G. , Gutierrez‐Ramos, J.‐C. , Williams, T. J. , & Rankin, S. M. (2003). Chemokines acting via CXCR2 and CXCR4 control the release of neutrophils from the bone marrow and their return following senescence. Immunity, 19(4), 583–593. 14563322 10.1016/s1074-7613(03)00263-2

[acel13700-bib-0122] Martini, E. , Krug, S. M. , Siegmund, B. , Neurath, M. F. , & Becker, C. (2017). Mend your fences: The epithelial barrier and its relationship with mucosal immunity in inflammatory bowel disease. Cellular and Molecular Gastroenterology and Hepatology, 4(1), 33–46. 28560287 10.1016/j.jcmgh.2017.03.007 PMC5439240

[acel13700-bib-0123] McKinley, E. T. , Sui, Y. , Al‐Kofahi, Y. , Millis, B. A. , Tyska, M. J. , Roland, J. T. , Santamaria‐Pang, A. , Ohland, C. L. , Jobin, C. , Franklin, J. L. , Lau, K. S. , Gerdes, M. J. , & Coffey, R. J. (2017). Optimized multiplex immunofluorescence single‐cell analysis reveals tuft cell heterogeneity. JCI Insight, 2(11), e93487. 10.1172/jci.insight.93487 PMC5453701 28570279

[acel13700-bib-0124] Mdawar, B. , Ghossoub, E. , & Khoury, R. (2020). Selective serotonin reuptake inhibitors and Alzheimer's disease. Neural Regeneration Research, 15(1), 41–46. 31535641 10.4103/1673-5374.264445 PMC6862425

[acel13700-bib-0125] Meyer‐Hoffert, U. , Hornef, M. W. , Henriques‐Normark, B. , Axelsson, L. G. , Midtvedt, T. , Pütsep, K. , & Andersson, M. (2008). Secreted enteric antimicrobial activity localises to the mucus surface layer. Gut, 57(6), 764–771. 18250125 10.1136/gut.2007.141481

[acel13700-bib-0126] Miller, C. N. , Proekt, I. , von Moltke, J. , Wells, K. L. , Rajpurkar, A. R. , Wang, H. , Rattay, K. , Khan, I. S. , Metzger, T. C. , Pollack, J. L. , Fries, A. C. , Lwin, W. W. , Wigton, E. J. , Parent, A. V. , Kyewski, B. , Erle, D. J. , Hogquist, K. A. , Steinmetz, L. M. , Locksley, R. M. , & Anderson, M. S. (2018). Thymic tuft cells promote an IL‐4‐enriched medulla and shape thymocyte development. Nature, 559(7715), 627–631. 30022164 10.1038/s41586-018-0345-2 PMC6062473

[acel13700-bib-0127] Mirpuri, J. , Raetz, M. , Sturge, C. R. , Wilhelm, C. L. , Benson, A. , Savani, R. C. , Hooper, L. V. , & Yarovinsky, F. (2014). Proteobacteria‐specific IgA regulates maturation of the intestinal microbiota. Gut Microbes, 5(1), 28–39. 24637807 10.4161/gmic.26489 PMC4049932

[acel13700-bib-0128] Molony, R. D. , Nguyen, J. T. , Kong, Y. , Montgomery, R. R. , Shaw, A. C. , & Iwasaki, A. (2017). Aging impairs both primary and secondary RIG‐I signaling for interferon induction in human monocytes. Science Signaling, 10(509), eaan2392. 29233916 10.1126/scisignal.aan2392 PMC6429941

[acel13700-bib-0129] Moorefield, E. C. , Andres, S. F. , Blue, R. E. , van Landeghem, L. , Mah, A. T. , Santoro, M. A. , & Ding, S. (2017). Aging effects on intestinal homeostasis associated with expansion and dysfunction of intestinal epithelial stem cells. Aging, 9(8), 1898–1915. 28854151 10.18632/aging.101279 PMC5611984

[acel13700-bib-0130] Moretto, M. M. , Lawlor, E. M. , & Khan, I. A. (2008). Aging mice exhibit a functional defect in mucosal dendritic cell response against an intracellular pathogen. Journal of Immunology, 181(11), 7977–7984. 10.4049/jimmunol.181.11.7977 PMC2676144 19017989

[acel13700-bib-0131] Mossad, O. , & Blank, T. (2021). Getting on in old age: How the gut microbiota interferes with brain innate immunity. Frontiers in Cellular Neuroscience, 15, 698126. 34295223 10.3389/fncel.2021.698126 PMC8290125

[acel13700-bib-0132] Motta, M. , Malaguarnera, M. , Ferrari, E. , Mauro, V. N. , Ferrucci, L. , Rapisarda, R. , Tomasello, F. B. , Basile, G. , Ferlito, L. , Passamonte, M. , & Bennati, E. (2007). Genealogy of centenarians and their relatives: a study of 12 families. Archives of Gerontology and Geriatrics, 45(1), 97–102. 17196681 10.1016/j.archger.2006.10.004 PMC2646091

[acel13700-bib-0133] Müller, L. , Di Benedetto, S. , & Pawelec, G. (2019). The immune system and its dysregulation with aging. Sub‐Cellular Biochemistry, 91, 21–43. 30888648 10.1007/978-981-13-3681-2_2

[acel13700-bib-0134] Nadjsombati, M. S. , McGinty, J. W. , Lyons‐Cohen, M. R. , Jaffe, J. B. , DiPeso, L. , Schneider, C. , Miller, C. N. , Pollack, J. L. , Nagana Gowda, G. A. , Fontana, M. F. , Erle, D. J. , Anderson, M. S. , Locksley, R. M. , Raftery, D. , & von Moltke, J. (2018). Detection of succinate by intestinal tuft cells triggers a type 2 innate immune circuit. Immunity, 49(1), 33–41.e7. 30021144 10.1016/j.immuni.2018.06.016 PMC6084797

[acel13700-bib-0135] Nagatake, T. , Fujita, H. , Minato, N. , & Hamazaki, Y. (2014). Enteroendocrine cells are specifically marked by cell surface expression of claudin‐4 in mouse small intestine. PLoS One, 9(6), e90638. 24603700 10.1371/journal.pone.0090638 PMC3948345

[acel13700-bib-0136] Nair, M. G. , Guild, K. J. , Du, Y. , Zaph, C. , Yancopoulos, G. D. , Valenzuela, D. M. , Murphy, A. , Stevens, S. , Karow, M. , & Artis, D. (2008). Goblet cell‐derived resistin‐like molecule beta augments CD4^+^ T cell production of IFN‐gamma and infection‐induced intestinal inflammation. Journal of Immunology, 181(7), 4709–4715. 10.4049/jimmunol.181.7.4709 PMC2819319 18802073

[acel13700-bib-0137] Nalapareddy, K. , Nattamai, K. J. , Kumar, R. S. , Karns, R. , Wikenheiser‐Brokamp, K. A. , Sampson, L. L. , Mahe, M. M. , Sundaram, N. , Yacyshyn, M. B. , Yacyshyn, B. , Helmrath, M. A. , Zheng, Y. , & Geiger, H. (2017). Canonical Wnt signaling ameliorates aging of intestinal stem cells. Cell Reports, 18(11), 2608–2621. 28297666 10.1016/j.celrep.2017.02.056 PMC5987258

[acel13700-bib-0138] Nicoletti, C. (2015). Age‐associated changes of the intestinal epithelial barrier: Local and systemic implications. Expert Review of Gastroenterology & Hepatology, 9(12), 1467–1469. 26395417 10.1586/17474124.2015.1092872

[acel13700-bib-0139] Nishida, A. , Inoue, R. , Inatomi, O. , Bamba, S. , Naito, Y. , & Andoh, A. (2018). Gut microbiota in the pathogenesis of inflammatory bowel disease. Clinical Journal of Gastroenterology, 11(1), 1–10. 29285689 10.1007/s12328-017-0813-5

[acel13700-bib-0140] Niwa, Y. , Kasama, T. , Miyachi, Y. , & Kanoh, T. (1989). Neutrophil chemotaxis, phagocytosis and parameters of reactive oxygen species in human aging: cross‐sectional and longitudinal studies. Life Sciences, 44(22), 1655–1664. 2733545 10.1016/0024-3205(89)90482-7

[acel13700-bib-0141] Nomellini, V. , Brubaker, A. L. , Mahbub, S. , Palmer, J. L. , Gomez, C. R. , & Kovacs, E. J. (2012). Dysregulation of neutrophil CXCR2 and pulmonary endothelial ICAM‐1 promotes age‐related pulmonary inflammation. Aging and Disease, 3(3), 234–247. 22724082 PMC3375080

[acel13700-bib-0142] Nyström, E. E. L. , Martinez‐Abad, B. , Arike, L. , Birchenough, G. M. H. , Nonnecke, E. B. , Castillo, P. A. , Svensson, F. , Bevins, C. L. , Hansson, G. C. , & Johansson, M. E. V. (2021). An intercrypt subpopulation of goblet cells is essential for colonic mucus barrier function. Science (New York, N.Y.), 372(6539), eabb1590. 10.1126/science.abb1590 PMC8542866 33859001

[acel13700-bib-0143] Nyugen, J. , Agrawal, S. , Gollapudi, S. , & Gupta, S. (2010). Impaired functions of peripheral blood monocyte subpopulations in aged humans. Journal of Clinical Immunology, 30(6), 806–813. 20703784 10.1007/s10875-010-9448-8 PMC2970801

[acel13700-bib-0144] Odamaki, T. , Kato, K. , Sugahara, H. , Hashikura, N. , Takahashi, S. , Xiao, J. Z. , Abe, F. , & Osawa, R. (2016). Age‐related changes in gut microbiota composition from newborn to centenarian: a cross‐sectional study. BMC Microbiology, 16, 90. 27220822 10.1186/s12866-016-0708-5 PMC4879732

[acel13700-bib-0145] Ogawa, K. , Suzuki, K. , Okutsu, M. , Yamazaki, K. , & Shinkai, S. (2008). The association of elevated reactive oxygen species levels from neutrophils with low‐grade inflammation in the elderly. Immunity & Ageing, 5(1), 13. 18950479 10.1186/1742-4933-5-13 PMC2582223

[acel13700-bib-0146] Ornelas, A. , Dowdell, A. S. , Lee, J. S. , & Colgan, S. P. (2022). Microbial metabolite regulation of epithelial cell‐cell interactions and barrier function. Cell, 11(6), 944. 10.3390/cells11060944 PMC8946845 35326394

[acel13700-bib-0147] Orsini, G. , Legitimo, A. , Failli, A. , Massei, F. , Biver, P. , & Consolini, R. (2012). Enumeration of human peripheral blood dendritic cells throughout the life. International Immunology, 24(6), 347–356. 22345276 10.1093/intimm/dxs006

[acel13700-bib-0148] O'Toole, P. W. , & Jeffery, I. B. (2015). Gut microbiota and aging. Science, 350(6265), 1214–1215. 26785481 10.1126/science.aac8469

[acel13700-bib-0149] Pang, W. W. , Price, E. A. , Sahoo, D. , Beerman, I. , Maloney, W. J. , Rossi, D. J. , Schrier, S. L. , & Weissman, I. L. (2011). Human bone marrow hematopoietic stem cells are increased in frequency and myeloid‐biased with age. Proceedings of the National Academy of Sciences of the United States of America, 108(50), 20012–20017. 22123971 10.1073/pnas.1116110108 PMC3250139

[acel13700-bib-0150] Parker, A. , Romano, S. , Ansorge, R. , Aboelnour, A. , le Gall, G. , Savva, G. M. , Pontifex, M. G. , Telatin, A. , Baker, D. , Jones, E. , Vauzour, D. , Rudder, S. , Blackshaw, L. A. , Jeffery, G. , & Carding, S. R. (2022). Fecal microbiota transfer between young and aged mice reverses hallmarks of the aging gut, eye, and brain. Microbiome, 10(1), 68. 35501923 10.1186/s40168-022-01243-w PMC9063061

[acel13700-bib-0151] Pépin, J. , Valiquette, L. , & Cossette, B. (2005). Mortality attributable to nosocomial *Clostridium difficile*‐associated disease during an epidemic caused by a hypervirulent strain in Quebec. CMAJ, 173(9), 1037–1042. 16179431 10.1503/cmaj.050978 PMC1266326

[acel13700-bib-0152] Perls, T. T. , Wilmoth, J. , Levenson, R. , Drinkwater, M. , Cohen, M. , Bogan, H. , Joyce, E. , Brewster, S. , Kunkel, L. , & Puca, A. (2002). Life‐long sustained mortality advantage of siblings of centenarians. Proceedings of the National Academy of Sciences of the United States of America, 99(12), 8442–8447. 12060785 10.1073/pnas.122587599 PMC123086

[acel13700-bib-0153] Pertovaara, M. , Raitala, A. , Lehtimäki, T. , Karhunen, P. J. , Oja, S. S. , Jylhä, M. , Hervonen, A. , & Hurme, M. (2006). Indoleamine 2,3‐dioxygenase activity in nonagenarians is markedly increased and predicts mortality. Mechanisms of Ageing and Development, 127(5), 497–499. 16513157 10.1016/j.mad.2006.01.020

[acel13700-bib-0154] Peterson, L. W. , & Artis, D. (2014). Intestinal epithelial cells: regulators of barrier function and immune homeostasis. Nature Reviews Immunology, 14(3), 141–153. 10.1038/nri3608 24566914

[acel13700-bib-0155] Politis, M. , & Niccolini, F. (2015). Serotonin in Parkinson's disease. Behavioural Brain Research, 277, 136–145. 25086269 10.1016/j.bbr.2014.07.037

[acel13700-bib-0156] Powell, D. N. , Swimm, A. , Sonowal, R. , Bretin, A. , Gewirtz, A. T. , Jones, R. M. , & Kalman, D. (2020). Indoles from the commensal microbiota act via the AHR and IL‐10 to tune the cellular composition of the colonic epithelium during aging. Proceedings of the National Academy of Sciences of the United States of America, 117(35), 21519–21526. 32817517 10.1073/pnas.2003004117 PMC7474656

[acel13700-bib-0157] Qi, Y. , Goel, R. , Kim, S. , Richards, E. M. , Carter, C. S. , Pepine, C. J. , Raizada, M. K. , & Buford, T. W. (2017). Intestinal permeability biomarker zonulin is elevated in healthy aging. Journal of the American Medical Directors Association, 18(9), 810.e1–810.e4. 10.1016/j.jamda.2017.05.018 PMC5581307 28676292

[acel13700-bib-0158] Ragonnaud, E. , & Biragyn, A. (2021). Gut microbiota as the key controllers of “healthy” aging of elderly people. Immunity & Ageing, 18(1), 2. 33397404 10.1186/s12979-020-00213-w PMC7784378

[acel13700-bib-0159] Ramos, C. , Gibson, G. R. , Walton, G. E. , Magistro, D. , Kinnear, W. , & Hunter, K. (2022). Systematic review of the effects of exercise and physical activity on the gut microbiome of older adults. Nutrients, 14(3), 674. 35277033 10.3390/nu14030674 PMC8837975

[acel13700-bib-0160] Rampelli, S. , Candela, M. , Turroni, S. , Biagi, E. , Pflueger, M. , Wolters, M. , Ahrens, W. , & Brigidi, P. (2016). Microbiota and lifestyle interactions through the lifespan. Trends in Food Science & Technology, 57, 265–272.

[acel13700-bib-0161] Reboldi, A. , & Cyster, J. G. (2016). Peyer's patches: organizing B‐cell responses at the intestinal frontier. Immunological Reviews, 271(1), 230–245. 27088918 10.1111/imr.12400 PMC4835804

[acel13700-bib-0162] Renshaw, M. , Rockwell, J. , Engleman, C. , Gewirtz, A. , Katz, J. , & Sambhara, S. (2002). Cutting edge: impaired Toll‐like receptor expression and function in aging. Journal of Immunology, 169(9), 4697–4701. 10.4049/jimmunol.169.9.4697 12391175

[acel13700-bib-0163] Rhee, S. H. , Pothoulakis, C. , & Mayer, E. A. (2009). Principles and clinical implications of the brain‐gut‐enteric microbiota axis. Nature Reviews. Gastroenterology & Hepatology, 6(5), 306–314. 19404271 10.1038/nrgastro.2009.35 PMC3817714

[acel13700-bib-0164] Rios, D. , Wood, M. B. , Li, J. , Chassaing, B. , Gewirtz, A. T. , & Williams, I. R. (2016). Antigen sampling by intestinal M cells is the principal pathway initiating mucosal IgA production to commensal enteric bacteria. Mucosal Immunology, 9(4), 907–916. 26601902 10.1038/mi.2015.121 PMC4917673

[acel13700-bib-0165] Rogier, R. , Ederveen, T. H. A. , Boekhorst, J. , Wopereis, H. , Scher, J. U. , Manasson, J. , Frambach, S. J. C. M. , Knol, J. , Garssen, J. , van der Kraan, P. , Koenders, M. I. , van den Berg, W. , van Hijum, S. , & Abdollahi‐Roodsaz, S. (2017). Aberrant intestinal microbiota due to IL‐1 receptor antagonist deficiency promotes IL‐17‐ and TLR4‐dependent arthritis. Microbiome, 5(1), 63. 28645307 10.1186/s40168-017-0278-2 PMC5481968

[acel13700-bib-0166] Rossi, D. J. , Bryder, D. , Zahn, J. M. , Ahlenius, H. , Sonu, R. , Wagers, A. J. , & Weissman, I. L. (2005). Cell intrinsic alterations underlie hematopoietic stem cell aging. Proceedings of the National Academy of Sciences of the United States of America, 102(26), 9194–9199. 15967997 10.1073/pnas.0503280102 PMC1153718

[acel13700-bib-0167] Rübe, C. E. , Fricke, A. , Widmann, T. A. , Fürst, T. , Madry, H. , Pfreundschuh, M. , & Rübe, C. (2011). Accumulation of DNA damage in hematopoietic stem and progenitor cells during human aging. PLoS One, 6(3), e17487. 21408175 10.1371/journal.pone.0017487 PMC3049780

[acel13700-bib-0168] Ruiz‐Ruiz, S. , Sanchez‐Carrillo, S. , Ciordia, S. , Mena, M. C. , Méndez‐García, C. , Rojo, D. , Bargiela, R. , Zubeldia‐Varela, E. , Martínez‐Martínez, M. , Barbas, C. , Ferrer, M. , & Moya, A. (2020). Functional microbiome deficits associated with ageing: Chronological age threshold. Aging Cell, 19(1), e13063. 31730262 10.1111/acel.13063 PMC6974723

[acel13700-bib-0169] Salazar, N. , Arboleya, S. , Fernández‐Navarro, T. , de los Reyes‐Gavilán, C. G. , Gonzalez, S. , & Gueimonde, M. (2019). Age‐associated changes in gut microbiota and dietary components related with the immune system in adulthood and old age: A cross‐sectional study. Nutrients, 11(8), 1765. 10.3390/nu11081765 PMC6722604 31370376

[acel13700-bib-0170] Salzman, N. H. , Hung, K. , Haribhai, D. , Chu, H. , Karlsson‐Sjöberg, J. , Amir, E. , Teggatz, P. , Barman, M. , Hayward, M. , Eastwood, D. , Stoel, M. , Zhou, Y. , Sodergren, E. , Weinstock, G. M. , Bevins, C. L. , Williams, C. B. , & Bos, N. A. (2010). Enteric defensins are essential regulators of intestinal microbial ecology. Nature Immunology, 11(1), 76–83. 19855381 10.1038/ni.1825 PMC2795796

[acel13700-bib-0171] San Hernandez, A. M. , Singh, C. , Valero, D. J. , Nisar, J. , Trujillo Ramirez, J. I. , Kothari, K. K. , Isola, S. , & Gordon, D. K. (2020). Multiple sclerosis and serotonin: Potential therapeutic applications. Cureus, 12(11), e11293. 33274166 10.7759/cureus.11293 PMC7707915

[acel13700-bib-0172] Sato, Y. , Atarashi, K. , Plichta, D. R. , Arai, Y. , Sasajima, S. , Kearney, S. M. , Suda, W. , Takeshita, K. , Sasaki, T. , Okamoto, S. , Skelly, A. N. , Okamura, Y. , Vlamakis, H. , Li, Y. , Tanoue, T. , Takei, H. , Nittono, H. , Narushima, S. , Irie, J. , … Honda, K. (2021). Novel bile acid biosynthetic pathways are enriched in the microbiome of centenarians. Nature, 599(7885), 458–464. 34325466 10.1038/s41586-021-03832-5

[acel13700-bib-0173] Schneider, C. (2021). Tuft cell integration of luminal states and interaction modules in tissues. Pflügers Archiv – European Journal of Physiology, 473(11), 1713–1722. 34635955 10.1007/s00424-021-02630-2 PMC8528756

[acel13700-bib-0174] Sepp, E. , Smidt, I. , Rööp, T. , Štšepetova, J. , Kõljalg, S. , Mikelsaar, M. , Soidla, I. , Ainsaar, M. , Kolk, H. , Vallas, M. , Jaagura, M. , & Mändar, R. (2022). Comparative analysis of gut microbiota in centenarians and young people: Impact of eating habits and childhood living environment. Frontiers in Cellular and Infection Microbiology, 12, 851404. 35372105 10.3389/fcimb.2022.851404 PMC8965453

[acel13700-bib-0175] Shaik‐Dasthagirisaheb, Y. B. , Kantarci, A. , & Gibson, F. C., 3rd . (2010). Immune response of macrophages from young and aged mice to the oral pathogenic bacterium *Porphyromonas gingivalis* . Immunity & Ageing: I & A, 7, 15. 21114831 10.1186/1742-4933-7-15 PMC3001696

[acel13700-bib-0176] Shaw, A. C. , Goldstein, D. R. , & Montgomery, R. R. (2013). Age‐dependent dysregulation of innate immunity. Nature Reviews Immunology, 13(12), 875–887. 10.1038/nri3547 PMC4096436 24157572

[acel13700-bib-0177] Shimada, Y. , Hayashi, M. , Nagasaka, Y. , Ohno‐Iwashita, Y. , & Inomata, M. (2009). Age‐associated up‐regulation of a negative co‐stimulatory receptor PD‐1 in mouse CD4^+^ T cells. Experimental Gerontology, 44(8), 517–522. 19457448 10.1016/j.exger.2009.05.003

[acel13700-bib-0178] Shin, J. H. , Gao, Y. , Moore, J. H., 2nd , Bolick, D. T. , Kolling, G. L. , Wu, M. , & Warren, C. A. (2018). Innate immune response and outcome of *Clostridium difficile* infection are dependent on fecal bacterial composition in the aged host. The Journal of Infectious Diseases, 217(2), 188–197. 28968660 10.1093/infdis/jix414 PMC5853981

[acel13700-bib-0179] Shukla, A. K. , Johnson, K. , & Giniger, E. (2021). Common features of aging fail to occur in *Drosophila* raised without a bacterial microbiome. iScience, 24(7), 102703. 34235409 10.1016/j.isci.2021.102703 PMC8246586

[acel13700-bib-0180] Smith, G. S. , Barrett, F. S. , Joo, J. H. , Nassery, N. , Savonenko, A. , Sodums, D. J. , Marano, C. M. , Munro, C. A. , Brandt, J. , Kraut, M. A. , Zhou, Y. , Wong, D. F. , & Workman, C. I. (2017). Molecular imaging of serotonin degeneration in mild cognitive impairment. Neurobiology of Disease, 105, 33–41. 28511918 10.1016/j.nbd.2017.05.007 PMC5663212

[acel13700-bib-0181] Smith, P. M. , Howitt, M. R. , Panikov, N. , Michaud, M. , Gallini, C. A. , Bohlooly‐Y, M. , Glickman, J. N. , & Garrett, W. S. (2013). The microbial metabolites, short‐chain fatty acids, regulate colonic Treg cell homeostasis. Science, 341(6145), 569–573. 23828891 10.1126/science.1241165 PMC3807819

[acel13700-bib-0182] Sonowal, R. , Swimm, A. , Sahoo, A. , Luo, L. , Matsunaga, Y. , Wu, Z. , Bhingarde, J. A. , Ejzak, E. A. , Ranawade, A. , Qadota, H. , Powell, D. N. , Capaldo, C. T. , Flacker, J. M. , Jones, R. M. , Benian, G. M. , & Kalman, D. (2017). Indoles from commensal bacteria extend healthspan. Proceedings of the National Academy of Sciences of the United States of America, 114(36), E7506–E7515. 28827345 10.1073/pnas.1706464114 PMC5594673

[acel13700-bib-0183] Sovran, B. , Hugenholtz, F. , Elderman, M. , van Beek, A. , Graversen, K. , Huijskes, M. , Boekschoten, M. V. , Savelkoul, H. F. J. , de Vos, P. , Dekker, J. , & Wells, J. M. (2019). Age‐associated impairment of the mucus barrier function is associated with profound changes in microbiota and immunity. Scientific Reports, 9(1), 1437. 30723224 10.1038/s41598-018-35228-3 PMC6363726

[acel13700-bib-0184] Srinivasan, T. , Than, E. B. , Bu, P. , Tung, K. L. , Chen, K. Y. , Augenlicht, L. , Lipkin, S. M. , & Shen, X. (2016). Notch signalling regulates asymmetric division and inter‐conversion between lgr5 and bmi1 expressing intestinal stem cells. Scientific Reports, 6(1), 26069. 27181744 10.1038/srep26069 PMC4867651

[acel13700-bib-0185] Stebegg, M. , Silva‐Cayetano, A. , Innocentin, S. , Jenkins, T. P. , Cantacessi, C. , Gilbert, C. , & Linterman, M. A. (2019). Heterochronic faecal transplantation boosts gut germinal centres in aged mice. Nature Communications, 10(1), 2443. 10.1038/s41467-019-10430-7 PMC6547660 31164642

[acel13700-bib-0186] Sternini, C. , Anselmi, L. , & Rozengurt, E. (2008). Enteroendocrine cells: a site of 'taste' in gastrointestinal chemosensing. Current Opinion in Endocrinology, Diabetes, and Obesity, 15(1), 73–78. 18185066 10.1097/MED.0b013e3282f43a73 PMC2943060

[acel13700-bib-0187] Stevenson, B. R. , Siliciano, J. D. , Mooseker, M. S. , & Goodenough, D. A. (1986). Identification of ZO‐1: a high molecular weight polypeptide associated with the tight junction (zonula occludens) in a variety of epithelia. The Journal of Cell Biology, 103(3), 755–766. 3528172 10.1083/jcb.103.3.755 PMC2114282

[acel13700-bib-0188] Sturgeon, C. , & Fasano, A. (2016). Zonulin, a regulator of epithelial and endothelial barrier functions, and its involvement in chronic inflammatory diseases. Tissue Barriers, 4(4), e1251384. 28123927 10.1080/21688370.2016.1251384 PMC5214347

[acel13700-bib-0189] Sun, R. , Xu, C. , Feng, B. , Gao, X. , & Liu, Z. (2021). Critical roles of bile acids in regulating intestinal mucosal immune responses. Therapeutic Advances in Gastroenterology, 14, 17562848211018098. 34104213 10.1177/17562848211018098 PMC8165529

[acel13700-bib-0190] Svendsen, B. , Pedersen, J. , Albrechtsen, N. J. , Hartmann, B. , Toräng, S. , Rehfeld, J. F. , Poulsen, S. S. , & Holst, J. J. (2015). An analysis of cosecretion and coexpression of gut hormones from male rat proximal and distal small intestine. Endocrinology, 156(3), 847–857. 25535831 10.1210/en.2014-1710

[acel13700-bib-0191] Taupin, D. R. , Kinoshita, K. , & Podolsky, D. K. (2000). Intestinal trefoil factor confers colonic epithelial resistance to apoptosis. Proceedings of the National Academy of Sciences of the United States of America, 97(2), 799–804. 10639160 10.1073/pnas.97.2.799 PMC15411

[acel13700-bib-0192] Thevaranjan, N. , Puchta, A. , Schulz, C. , Naidoo, A. , Szamosi, J. C. , Verschoor, C. P. , Loukov, D. , Schenck, L. P. , Jury, J. , Foley, K. P. , Schertzer, J. D. , Larché, M. J. , Davidson, D. J. , Verdú, E. F. , Surette, M. G. , & DME, B. (2017). Age‐associated microbial dysbiosis promotes intestinal permeability, systemic inflammation, and macrophage dysfunction. Cell Host & Microbe, 21(4), 455–66.e4. 28407483 10.1016/j.chom.2017.03.002 PMC5392495

[acel13700-bib-0193] Thim, L. , Madsen, F. , & Poulsen, S. S. (2002). Effect of trefoil factors on the viscoelastic properties of mucus gels. European Journal of Clinical Investigation, 32(7), 519–527. 12153553 10.1046/j.1365-2362.2002.01014.x

[acel13700-bib-0194] Thomas, R. , Wang, W. , & Su, D. M. (2020). Contributions of age‐related thymic involution to immunosenescence and inflammaging. Immunity & Ageing: I & A, 17, 2. 31988649 10.1186/s12979-020-0173-8 PMC6971920

[acel13700-bib-0195] Tian, H. , Biehs, B. , Chiu, C. , Siebel, C. W. , Wu, Y. , Costa, M. , de Sauvage, F. J. , & Klein, O. D. (2015). Opposing activities of notch and wnt signaling regulate intestinal stem cells and gut homeostasis. Cell Reports, 11(1), 33–42. 25818302 10.1016/j.celrep.2015.03.007 PMC4394041

[acel13700-bib-0196] Toapanta, F. R. , & Ross, T. M. (2009). Impaired immune responses in the lungs of aged mice following influenza infection. Respiratory Research, 10(1), 112. 19922665 10.1186/1465-9921-10-112 PMC2785782

[acel13700-bib-0197] Tran, L. , & Greenwood‐Van Meerveld, B. (2013). Age‐associated remodeling of the intestinal epithelial barrier. The Journals of Gerontology: Series A, 68(9), 1045–1056. 10.1093/gerona/glt106 PMC3738030 23873964

[acel13700-bib-0198] Tremblay, S. , NML, C. , Grenier, G. , Duclos‐Lasnier, G. , Fortier, L. C. , Ilangumaran, S. , & Menendez, A. (2017). Ileal antimicrobial peptide expression is dysregulated in old age. Immunity & Ageing: I & A, 14, 19. 28855949 10.1186/s12979-017-0101-8 PMC5575895

[acel13700-bib-0199] Tuikhar, N. , Keisam, S. , Labala, R. K. , Imrat , Ramakrishnan, P. , Arunkumar, M. C. , Ahmed, G. , Biagi, E. , & Jeyaram, K. (2019). Comparative analysis of the gut microbiota in centenarians and young adults shows a common signature across genotypically non‐related populations. Mechanisms of Ageing and Development, 179, 23–35. 30738080 10.1016/j.mad.2019.02.001

[acel13700-bib-0200] Turner, J. R. (2009). Intestinal mucosal barrier function in health and disease. Nature Reviews Immunology, 9(11), 799–809. 10.1038/nri2653 19855405

[acel13700-bib-0201] Uchida, R. , Saito, Y. , Nogami, K. , Kajiyama, Y. , Suzuki, Y. , Kawase, Y. , Nakaoka, T. , Muramatsu, T. , Kimura, M. , & Saito, H. (2018). Epigenetic silencing of Lgr5 induces senescence of intestinal epithelial organoids during the process of aging. NPJ Aging and Mechanisms of Disease, 4(1), 12. 10.1038/s41514-018-0031-5 PMC6279747 30534415

[acel13700-bib-0202] Umeda, K. , Ikenouchi, J. , Katahira‐Tayama, S. , Furuse, K. , Sasaki, H. , Nakayama, M. , Matsui, T. , Tsukita, S. , Furuse, M. , & Tsukita, S. (2006). ZO‐1 and ZO‐2 independently determine where claudins are polymerized in tight‐junction strand formation. Cell, 126(4), 741–754. 16923393 10.1016/j.cell.2006.06.043

[acel13700-bib-0203] Vaishnava, S. , Behrendt, C. L. , Ismail, A. S. , Eckmann, L. , & Hooper, L. V. (2008). Paneth cells directly sense gut commensals and maintain homeostasis at the intestinal host‐microbial interface. Proceedings of the National Academy of Sciences of the United States of America, 105(52), 20858–20863. 19075245 10.1073/pnas.0808723105 PMC2603261

[acel13700-bib-0204] Vaishnava, S. , Yamamoto, M. , Severson, K. M. , Ruhn, K. A. , Yu, X. , Koren, O. , Ley, R. , Wakeland, E. K. , & Hooper, L. V. (2011). The antibacterial lectin RegIIIgamma promotes the spatial segregation of microbiota and host in the intestine. Science (New York, N.Y.), 334(6053), 255–258. 10.1126/science.1209791 PMC3321924 21998396

[acel13700-bib-0205] van Beek, A. A. , Sovran, B. , Hugenholtz, F. , Meijer, B. , Hoogerland, J. A. , Mihailova, V. , van der Ploeg, C. , Belzer, C. , Boekschoten, M. V. , Hoeijmakers, J. H. , Vermeij, W. P. , de Vos, P. , Wells, J. M. , Leenen, P. J. , Nicoletti, C. , Hendriks, R. W. , & Savelkoul, H. F. (2016). Supplementation with *Lactobacillus plantarum* WCFS1 prevents decline of mucus barrier in colon of accelerated aging Ercc1(−/Δ7) mice. Frontiers in Immunology, 7, 408. 27774093 10.3389/fimmu.2016.00408 PMC5054004

[acel13700-bib-0206] van der Lugt, B. , van Beek, A. A. , Aalvink, S. , Meijer, B. , Sovran, B. , Vermeij, W. P. , RMC, B. , de Vos, W. M. , HFJ, S. , Steegenga, W. T. , & Belzer, C. (2019). Akkermansia muciniphila ameliorates the age‐related decline in colonic mucus thickness and attenuates immune activation in accelerated aging Ercc1−/Δ7 mice. Immunity & Ageing, 16(1), 6. 30899315 10.1186/s12979-019-0145-z PMC6408808

[acel13700-bib-0207] van Duin, D. , Mohanty, S. , Thomas, V. , Ginter, S. , Montgomery, R. R. , Fikrig, E. , Allore, H. G. , Medzhitov, R. , & Shaw, A. C. (2007). Age‐associated defect in human TLR‐1/2 function. Journal of Immunology, Virus Research and Experimental Chemotherapy, 178(2), 970–975. 10.4049/jimmunol.178.2.970 17202359

[acel13700-bib-0208] Van Epps, P. , Oswald, D. , Higgins, P. A. , Hornick, T. R. , Aung, H. , Banks, R. E. , Wilson, B. M. , Burant, C. , Graventstein, S. , & Canaday, D. H. (2016). Frailty has a stronger association with inflammation than age in older veterans. Immunity & Ageing, 13(1), 27. 27777599 10.1186/s12979-016-0082-z PMC5069820

[acel13700-bib-0209] von Moltke, J. , Ji, M. , Liang, H. E. , & Locksley, R. M. (2016). Tuft‐cell‐derived IL‐25 regulates an intestinal ILC2‐epithelial response circuit. Nature, 529(7585), 221–225. 26675736 10.1038/nature16161 PMC4830391

[acel13700-bib-0210] Walrath, T. , Dyamenahalli, K. U. , Hulsebus, H. J. , McCullough, R. L. , Idrovo, J.‐P. , Boe, D. M. , McMahan, R. , & Kovacs, E. J. (2021). Age‐related changes in intestinal immunity and the microbiome. Journal of Leukocyte Biology, 109(6), 1045–1061. 33020981 10.1002/JLB.3RI0620-405RR PMC8139861

[acel13700-bib-0211] Walton, K. L. , He, J. , Kelsall, B. L. , Sartor, R. B. , & Fisher, N. C. (2006). Dendritic cells in germ‐free and specific pathogen‐free mice have similar phenotypes and in vitro antigen presenting function. Immunology Letters, 102(1), 16–24. 16105690 10.1016/j.imlet.2005.07.001

[acel13700-bib-0212] Wenisch, C. , Patruta, S. , Daxböck, F. , Krause, R. , & Hörl, W. (2000). Effect of age on human neutrophil function. Journal of Leukocyte Biology, 67(1), 40–45. 10647996 10.1002/jlb.67.1.40

[acel13700-bib-0213] Wilmanski, T. , Diener, C. , Rappaport, N. , Patwardhan, S. , Wiedrick, J. , Lapidus, J. , Earls, J. C. , Zimmer, A. , Glusman, G. , Robinson, M. , Yurkovich, J. T. , Kado, D. M. , Cauley, J. A. , Zmuda, J. , Lane, N. E. , Magis, A. T. , Lovejoy, J. C. , Hood, L. , Gibbons, S. M. , … Price, N. D. (2021). Gut microbiome pattern reflects healthy ageing and predicts survival in humans. Nature Metabolism, 3(2), 274–286. 10.1038/s42255-021-00348-0 PMC8169080 33619379

[acel13700-bib-0214] Wilms, E. , Troost, F. J. , Elizalde, M. , Winkens, B. , de Vos, P. , Mujagic, Z. , Jonkers, D. M. A. E. , & Masclee, A. A. M. (2020). Intestinal barrier function is maintained with aging – A comprehensive study in healthy subjects and irritable bowel syndrome patients. Scientific Reports, 10(1), 475. 31949225 10.1038/s41598-019-57106-2 PMC6965102

[acel13700-bib-0215] Woodmansey, E. J. (2007). Intestinal bacteria and ageing. Journal of Applied Microbiology, 102(5), 1178–1186. 17448153 10.1111/j.1365-2672.2007.03400.x

[acel13700-bib-0216] Worthington, J. J. , Reimann, F. , & Gribble, F. M. (2018). Enteroendocrine cells‐sensory sentinels of the intestinal environment and orchestrators of mucosal immunity. Mucosal Immunology, 11(1), 3–20. 28853441 10.1038/mi.2017.73

[acel13700-bib-0217] Woudstra, T. D. , Drozdowski, L. A. , Wild, G. E. , Clandinin, M. T. , Agellon, L. B. , & Thomson, A. B. R. (2004). The age‐related decline in intestinal lipid uptake is associated with a reduced abundance of fatty acid‐binding protein. Lipids, 39(7), 603–610. 15588016 10.1007/s11745-004-1272-9

[acel13700-bib-0218] Wu, C. S. , Muthyala, S. D. V. , Klemashevich, C. , Ufondu, A. U. , Menon, R. , Chen, Z. , Devaraj, S. , Jayaraman, A. , & Sun, Y. (2021). Age‐dependent remodeling of gut microbiome and host serum metabolome in mice. Aging, 13(5), 6330–6345. 33612480 10.18632/aging.202525 PMC7993679

[acel13700-bib-0219] Wu, L. , Zeng, T. , Zinellu, A. , Rubino, S. , Kelvin, D. J. , & Carru, C. (2019). A cross‐sectional study of compositional and functional profiles of gut microbiota in Sardinian Centenarians. mSystems, 4(4), e00325‐19. 31289141 10.1128/mSystems.00325-19 PMC6616150

[acel13700-bib-0220] Xiao, Y. , Huang, X. , Zhao, Y. , Chen, F. , Sun, M. , Yang, W. , Chen, L. , Yao, S. , Peniche, A. , Dann, S. M. , Sun, J. , Golovko, G. , Fofanov, Y. , Miao, Y. , Liu, Z. , Chen, D. , & Cong, Y. (2019). Interleukin‐33 promotes REG3γ expression in intestinal epithelial cells and regulates gut microbiota. Cellular and Molecular Gastroenterology and Hepatology, 8(1), 21–36. 30831322 10.1016/j.jcmgh.2019.02.006 PMC6510930

[acel13700-bib-0221] Xu, C. , Zhu, H. , & Qiu, P. (2019). Aging progression of human gut microbiota. BMC Microbiology, 19(1), 236. 31660868 10.1186/s12866-019-1616-2 PMC6819604

[acel13700-bib-0222] Yoshimoto, S. , Mitsuyama, E. , Yoshida, K. , Odamaki, T. , & Xiao, J. Z. (2021). Enriched metabolites that potentially promote age‐associated diseases in subjects with an elderly‐type gut microbiota. Gut Microbes, 13(1), 1–11. 10.1080/19490976.2020.1865705 PMC7808425 33430687

[acel13700-bib-0223] Yu, Y. , Daly, D. M. , Adam, I. J. , Kitsanta, P. , Hill, C. J. , Wild, J. , Shorthouse, A. , Grundy, D. , & Jiang, W. (2016). Interplay between mast cells, enterochromaffin cells, and sensory signaling in the aging human bowel. Neurogastroenterology and Motility, 28(10), 1465–1479. 27206689 10.1111/nmo.12842 PMC5053273

[acel13700-bib-0224] Zhang, D. , Chen, G. , Manwani, D. , Mortha, A. , Xu, C. , Faith, J. J. , Burk, R. D. , Kunisaki, Y. , Jang, J. E. , Scheiermann, C. , Merad, M. , & Frenette, P. S. (2015). Neutrophil ageing is regulated by the microbiome. Nature, 525(7570), 528–532. 26374999 10.1038/nature15367 PMC4712631

[acel13700-bib-0225] Zhang, J. G. , Cong, B. , Li, Q. X. , Chen, H. Y. , Qin, J. , & Fu, L. H. (2011). Cholecystokinin octapeptide regulates lipopolysaccharide‐activated B cells co‐stimulatory molecule expression and cytokines production *in vitro* . Immunopharmacology and Immunotoxicology, 33(1), 157–163. 20536341 10.3109/08923973.2010.491079

[acel13700-bib-0226] Zhang, J. G. , Liu, J. X. , Jia, X. X. , Geng, J. , Yu, F. , & Cong, B. (2014). Cholecystokinin octapeptide regulates the differentiation and effector cytokine production of CD4^+^ T cells in vitro. International Immunopharmacology, 20(2), 307–315. 24704498 10.1016/j.intimp.2014.03.013

